# The Expanding Therapeutic Utility of Botulinum Neurotoxins

**DOI:** 10.3390/toxins10050208

**Published:** 2018-05-18

**Authors:** Elena Fonfria, Jacquie Maignel, Stephane Lezmi, Vincent Martin, Andrew Splevins, Saif Shubber, Mikhail Kalinichev, Keith Foster, Philippe Picaut, Johannes Krupp

**Affiliations:** 1 Ipsen Bioinnovation, 102 Park Drive, Milton Park, Abingdon, Oxfordshire OX14 4RY, UK; andrew.splevins@ipsen.com (A.S.); keith.foster@ipsen.com (K.F.); 2 Ipsen Innovation, 5 Avenue du Canada, 91940 Les Ulis, France; jacquie.maignel@ipsen.com (J.M.); stephane.lezmi@ipsen.com (S.L.); vincent.martin@ipsen.com (V.M.); mikhail.kalinichev@ipsen.com (M.K.); johannes.krupp@ipsen.com (J.K.); 3 Ipsen Biopharm Ltd., Wrexham Industrial Estate, 9 Ash Road, Wrexham LL13 9UF, UK; saif.shubber@ipsen.com; 4 Ipsen Bioscience, 650 Kendall Street, Cambridge, MA 02142, USA; philippe.picaut@ipsen.com

**Keywords:** new indications, formulation, delivery

## Abstract

Botulinum neurotoxin (BoNT) is a major therapeutic agent that is licensed in neurological indications, such as dystonia and spasticity. The BoNT family, which is produced in nature by clostridial bacteria, comprises several pharmacologically distinct proteins with distinct properties. In this review, we present an overview of the current therapeutic landscape and explore the diversity of BoNT proteins as future therapeutics. In recent years, novel indications have emerged in the fields of pain, migraine, overactive bladder, osteoarthritis, and wound healing. The study of biological effects distal to the injection site could provide future opportunities for disease-tailored BoNT therapies. However, there are some challenges in the pharmaceutical development of BoNTs, such as liquid and slow-release BoNT formulations; and, transdermal, transurothelial, and transepithelial delivery. Innovative approaches in the areas of formulation and delivery, together with highly sensitive analytical tools, will be key for the success of next generation BoNT clinical products.

## 1. Current Therapeutic Landscape

Based upon the use of neutralising antibodies, there are currently seven different serotypes of botulinum neurotoxin (BoNT) that have been reported, from BoNT A through to G. More recently, an eighth serotype has been identified at the protein sequence level, BoNT/X, although this has not yet been produced or characterised as a protein [1]. Over the last 15 years, it has also been recognised that within each serotype there are multiple sub-types, each with a unique protein sequence and a distinct molecular entity [2]. All BoNT serotypes and their subtypes inhibit the neurotransmitter release from nerve terminals through the prevention of acetylcholine release at the neuromuscular junction, which results in flaccid paralysis. Despite this, the intracellular target proteins, receptors, pharmacodynamic properties, and potencies vary substantially between BoNT serotypes.

Despite the great diversity of natural BoNTs and the fact that there are several manufacturers of BoNT for both aesthetic and therapeutic applications, to date, all of the commercially available BoNT products are serotype BoNT/A1, except for a single serotype BoNT/B product, Myobloc^®^/Neurobloc^®^. However, BoNT/B showed to be less potent in the clinic than had been anticipated based on its efficacy during animal studies. The reason for this has recently been identified as a residue difference within human synaptotagmin II, which is the protein receptor for BoNT/B [3,4]. The residue difference is within the binding recognition sequence of synaptotagmin II and renders human synaptotagmin II a lower affinity receptor for BoNT/B than in other species. The low affinity for human synaptotagmin II requires higher doses of toxin to be injected in order to achieve efficacy that is similar to BoNT/A, and this results in a different safety profile and a high rate (up to 18%) of neutralizing antibodies as compared to BoNT/A (0 to 5%) ([5]; FDA label). For this reason, the BoNT/B product is not widely used. The identification of the molecular explanation for BoNT/B having lower efficacy in humans has enabled the design of modified BoNT/B sequences that are able to bind human synaptotagmin II with higher affinity, and so to potentially have improved therapeutic characteristics [6].

The three most widely used and commercially available BoNT/A products are Dysport^®^ (abobotulinumtoxinA, Ipsen, Paris, France), Botox^®^ (onabotulinumtoxinA, Allergan, Dublin, Ireland), and Xeomin^®^ (incobotulinumtoxinA, Merz, Frankfurt am Main, Germany). While the mechanism of action of these three products is the same, there are differences between them, since the BoNT/A that is produced and purified is specific to each toxin manufacturer, and the final formulation is different for each product. For example, the potency units are different for each product, and they are not interchangeable [7]. The unit is defined as the mouse LD50 by the intraperitoneal route, and each company assesses these in its own proprietary assay. This means that a “Dysport^®^ unit” is not the same as a “Botox^®^ unit”, or yet again, a “Xeomin^®^ unit”. The clinically approved doses, which are measured in the respective units, are again, therefore, different between the products. The amount of human serum albumin also differs (highest in Xeomin^®^ [1 mg], when compared to Botox^®^ [500 µg], and lowest in Dysport^®^ [125 µg]), and most importantly, the amount of neurotoxin is different in each individual product (3.24 ng per 500 U vial for Dysport^®^; 0.73 ng per 100 U vial for Botox^®^; 0.44 ng per 100 U vial for Xeomin^®^ [8]), leading to potentially different efficacy profiles in humans.

An excellent review of commercially available BoNT products and their manufacture is provided in Pickett, 2014 [9]. The key aspects of the major BoNT products that are available in the United States and Europe are given in Table 1. Currently, all of the commercially available BoNT products are manufactured using native *Clostridium botulinum* as the production organism, although some companies are exploring the use of recombinant expression using *Escherichia coli*. This would allow a standardised and safer manufacturing process for BoNT/A and for other serotypes and genetically modified versions of the neurotoxin.

In the United States of America (USA) and Europe, both Dysport^®^ and Botox^®^ are approved for use in adult upper and lower limb spasticity, while Xeomin^®^ is only approved for use in adult upper limb spasticity (the Xeomin adult lower limb Phase 3 pivotal study did not meet its primary endpoint at the 400 U dose [NCT01464307, https://clinicaltrials.gov/]). Dysport^®^ is the only BoNT/A product approved in the USA for the use in children with lower limb spasticity. The Botox^®^ study is still ongoing, (NCT01603628), while the Xeomin^®^ pivotal Phase 3 study did not meet its primary endpoint (NCT01893411).

Over the last two decades, the localized efficacy of BoNT/A, and its well tolerated safety profile, has resulted in significant growth of on-label use across multiple therapeutic and aesthetic indications. BoNT/As are well tolerated, which has also favoured significant growth in its empirical/off-label use in a variety of movement, ophthalmologic, gastrointestinal, urologic, orthopedic, dermatologic, secretory, and pain disorders.

BoNT is injected locally in skeletal muscles (on-label such as cervical dystonia, hemifascial spasm, blepharospasm, spasticity in adult and children; or, off-label such as writer cramp, tremors, spasmodic dysphonia), smooth muscles (on-label neurogenic detrusor overactivity, idiopathic bladder overactivity; or, off label such as bladder pain syndrome, detrusor sphincter dyssynergia) or exocrine gland hyperfunction (on-label sialorrhea, axillary hyperhidrosis; or, off-label, such as Frey’s syndrome, plantar/palmar hyperhidrosis). More recently, BoNT has been used in pain related disorders (on-label chronic migraine or off-label, such as osteoarthritis, neuropathic pain, lower back pain). In aesthetic, BoNT is used to treat multiple facial hyperkinetic lines that are related to striated muscles spasm (on-label glabellar lines, lateral canthal lines, front lines; or, off-label such as lateral eyebrow lift, nasal lines, mid and lower face, neck).

Currently available BoNT products have certain limitations. As the injection is local, there is the risk, although very low, for the toxin to diffuse locally in the vicinity of the tissue injected and induce unwanted effects, or more importantly, to spread far from the original injection site. These unwanted side effects are influenced by the injection technique, the dose, and the volume. The injection procedure can also be a limitation, as it necessitates the specific training for injectors and can be painful for patients, hence there is a need to develop novel formulations and delivery techniques, such as transdermal delivery or products with a longer duration of response (BoNT/A products are reinjected every three to four months in most indications, while an injection interval of six to nine months duration would be of clinical interest). Limitations of the current toxin products may also relate to vial size (3 to 10 mL) relative to some therapeutic indications where the injection volume can be 15 to 30 mL. In addition to these limitations, when seeking to expand the approved clinical indications for BoNT products, there is a strong need to assess the risk-benefit profile through well-designed research clinical trials.

## 2. Alternative Serotypes, Broadening the Therapeutic Landscape

Although only BoNT/A or BoNT/B serotype products are currently available as licenced clinical products, several other serotypes of BoNT have been explored as potential therapeutic agents in humans. The use of BoNT/F as an alternative serotype in patients who had become resistant to BoNT/A through antibody formation was first reported in 1992 [10]. Subsequent studies confirmed the clinical benefit of BoNT/F in dystonic patients, and revealed that it had a significantly shorter duration of response [11,12,13,14,15,16,17].

Another BoNT serotype, with an even shorter duration of response, is BoNT/E. A comparison of BoNT/E and BoNT/A in the *extensor digitorum brevis* muscle of human volunteers using compound muscle action potential (CMAP) to assess the efficacy that was demonstrated a much faster recovery following BoNT/E administration [18]. Surprisingly, in this study, when the *extensor digitorum brevis* was injected with a combination of both BoNT/A and E, recovery was similar to that observed with BoNT/E alone; this is not consistent with the findings in a number of animal experiments, nor with the understanding that BoNT/A light chain survives in the neuronal cytosol and results in ongoing paralysis, even after the BoNT/E has worn off [19,20,21]. Short acting BoNTs, such as E and F, have potential clinical applications in therapeutic areas where a significantly shorter duration of response (3–6 weeks) when compared to that of BoNT/A (3–4 months) is required, for example, in orthopedics and rehabilitation medicine [22]. Recently, a biotechnology company, Bonti, announced that it is developing a BoNT/E product, EB-001, in aesthetic and therapeutic indications. The first phase 2 study, with EB-001 in glabellar frown lines, demonstrated the safety and clinical efficacy for this indication. Bonti have also initiated a phase 2 clinical study to evaluate the safety and efficacy of EB-001 by intramuscular injection in reducing musculoskeletal pain in patients undergoing elective augmentation mammoplasty. Ipsen also have a recombinant BoNT/E in phase 1 clinical studies; this is the first recombinant botulinum neurotoxin ever to have entered clinical trials in humans.

In addition to its shorter duration of response, which offers therapeutic differentiation from existing BoNT products, preclinical studies also indicate that BoNT/E has additional properties that may open up novel therapeutic applications. Experimental studies in rats showed that BoNT/E that was injected into the hippocampus inhibited glutamate release and reduced both focal and generalised kainic acid-induced seizures [23]. BoNT/E also prevented neuronal loss and long term cognitive defects that were associated with kainic acid seizures, and reduced sensitivity to electrical stimulation of kindling, indicating an antiepileptogenic activity. The authors suggested that this activity is a result of selective inhibition of excitatory glutaminergic versus GABAergic transmission by BoNT/E attributable to preferential localisation of SNAP-25 in excitatory hippocampal neurons.

A third alternative to BoNT/A, which was explored for its potential therapeutic utility, is serotype BoNT/C. A comparison of the neuromuscular blockade induced by BoNT/C, assessed electrophysiologically in the *extensor digitorum brevis* muscle of human volunteers, showed an efficacy and duration of action that was very similar to BoNT/A [24]. The same authors reported that it was used to treat two patients with idiopathic facial hemispasm and one patient with blepharospasm with long lasting beneficial effects. BoNT/C injections, like BoNT/A injections, did not affect the motor neuron count in human volunteers, showing it to be well tolerated [25]. BoNT/C was successfully tested in a pilot series of BoNT/A non-responsive patients with focal dystonia (four with torticollis and two with blepharospasm) [26]. Patients did not develop secondary resistance to BoNT/C after chronic use. The activity of BoNT/C in BoNT/A-resistant patients was further confirmed in patients with cervical dystonia [27]. Despite this early clinical interest, as of yet, no commercial product based upon BoNT/C has been produced, possibly reflecting that it is not sufficiently differentiated from BoNT/A in its clinical characteristics.

In addition to the BoNT serotypes that are described above, potential differences between BoNT/A subtypes have also been explored. Comparison of BoNT/A subtypes 1 to 5 revealed distinct characteristics both in vitro and in vivo [28,29]. BoNT/A2 was more potent in vitro and in vivo compared to BoNT/A1 [28,30,31,32]. As BoNT/A2 was reported to enter neuronal cells faster than BoNT/A1, this was proposed to be responsible for the difference in potency [30]. It has also been suggested that a higher occupancy of the cellular receptors by BoNT/A2 as compared to BoNT/A1 is the underlying mechanism [33]. The crystal structure of BoNT/A2 bound to the luminal domain of its cognate protein receptor SV2C, has been recently resolved, showing that the mode of binding of BoNT/A2 to SV2C does not substantially differ from that of BoNT/A1 [34]. The intoxication symptoms in mice that were injected i.v. with 10^4^ units/mL of BoNT/A2 have been reported to differ from those in mice injected i.v. with 10^5^ units/mL of BoNT/A1 [29,35]. Using a twitch tension assay in mouse hemi-diaphragm and rat grip strength model it was proposed that BoNT/A2 was a more potent neuromuscular blocker and spread less to the contralateral limb than BoNT/A1 [32]. For further discussion of the differences in spread between BoNT/A1 and BoNT/A2, see Section 4.4. It has also been reported that BoNT/A2 is less immunogenic than BoNT/A1 [36], but the comparison compared a complexed form in the case of BoNT/A1 with purified BoNT/A2, so there is the potential that the differential presence of complexing proteins influenced the measured immunogenicity. The authors also claimed that BoNT/A2 was less susceptible to neutralisation by human antisera raised to BoNT/A1 complex toxoid vaccine. In healthy volunteers, BoNT/A2 caused a reduction in CMAP with a comparable onset and duration as onabotulinumtoxin A [37]. Subsequently, BoNT/A2 was compared to onabotulinumtoxin A in post-stroke spasticity. BoNT/A2 was reported to show higher efficacy and less spread, as measured by the hand grip of the unaffected side, than the A1 toxin [38]. Animal models have also shown that BoNT/A2 could be a promising therapeutic in Parkinson’s disease and inflammatory and neuropathic pain [39,40,41]. In 2016, Shionogi & Co. Ltd. (Osaka, Japan) announced a licence agreement with Tokoshima University for BoNT/A2, and is undertaking its global development as a novel BoNT therapeutic.

## 3. Novel Indications

### 3.1. Pain and Migraine

Pain is the most common reason for a patient to seek medical help, and it is seen by physicians as a symptom of an underlying medical condition [42]. While acute pain typically measures in days to weeks, chronic pain can persist from months to years, thus representing a major health-care issue with a serious impact on quality of life and significant socio-economic cost [43,44]. Chronic pain is classified as inflammatory (such as osteoarthritis, rheumatoid arthritis), neuropathic (such as diabetic, post-herpetic neuralgia), or dysfunctional (such as tension type headache, migraine, interstitial cystitis, irritable bowel syndrome) [45]. Current medication for acute and chronic pain includes opioids, cyclooxygenase inhibitors, acetaminophen, as well as several repositioned drugs, such as the antidepressant drug duloxetine and the antiepileptic drug pregabalin. While these drugs offer some pain relief, they are not consistently effective, often result in tolerance when being taken over a prolonged period and cause serious side-effects that hinder their use [46]. Therefore, there is a need for new pain therapeutics that can offer improved efficacy, reduced propensity to cause tolerance, and fewer side-effects. There is rapidly growing evidence that BoNT can offer an effective, long-lasting pain relief, and very few side-effects in a wide range of medical conditions.

The initial evidence of pain relief in response to BoNT treatment is linked to several serendipitous observations in clinical studies involving muscle hyperactivity and muscle pain in patients with spasticity, dystonia, and related conditions. While muscle relaxant effects may play a role in some of these conditions, there is growing evidence that they cannot account for all mechanisms mediating pain relief following BoNT treatment across a growing range of medical conditions. For example, even in conditions involving intramuscular-administered BoNT, effects in pain can precede and/or last longer in comparison to the muscle-relaxant effects [47,48]. Also, it is now well-accepted that, in addition to local uptake in the synaptic terminal, a distinct secondary uptake pathway results in retrograde transport of BoNT and its activity at distal sites [49]. The retrograde transport of BoNT from the site of uptake at the sensory neuron ending into the dorsal root ganglion and the spinal cord is believed to play a pivotal role for the activity of BoNT in pain [50,51]. In addition to neurons, BoNT can impact the functional activity of glial cells, such as Schwann cells and astrocytes, which suggests the presence of yet another mechanism of pain modulation by BoNT [52,53,54].

Since the initial discovery several decades ago, the field of therapeutic application of BoNT in pain has been growing rapidly, which was predominately led by observations and studies performed in clinical settings. Despite the rapid growth of the field, prophylactic treatment of chronic migraine is the only pain indication currently approved based on the outcome of two multi-center phase 3 studies [55] and their combined *post hoc* analysis [56]. Reflecting the recent grade A recommendation of the American Academy of Neurology, BoNT/A should be used in chronic migraine and should not be used (as ineffective) in episodic migraine [57]. Apart from these two migraine indications with clear recommendations on the use of BoNT/A, there are a number of other types of headaches where the evidence is not as clear, as both positive and negative clinical results have been obtained. For example, the effects of BoNT/A in tension-type headache, which is the most common primary headache and pain condition, have been evaluated in several open-label and randomized, placebo-controlled studies. BoNT/A injected into the pericranial muscles every three months during 18 months (in an open-label study) or only once (in a double-blind study), reduced the severity of headache, reduced pericranial muscle tenderness, and increased the number of headache-free days [58]. In contrast, BoNT/A failed to improve any measures of tension-type headache in two other placebo-controlled, double-blind clinical studies [59,60]. Interestingly, in another double-blind placebo-controlled study, no difference was seen between the BoNT/A and the placebo group on Day 60, but a significant number of patients reported a 50% decrease in headache days at Day 90 [61]. This suggests that, at least with tension-type headache, longer post-treatment periods need to be evaluated in order to see the effect. Similar discrepancies in the efficacy of BoNT in pain across clinical studies have been seen in other indications, such as osteopathic pain [62].

What makes it difficult for a given pain indication to interpret and to compare the results across clinical studies, is the fact that there is no consistency in the experimental variables used, such as sites and routes of injection, number of injections needed, doses, administration regimens (single vs. repeated), etc. Back-translating the human findings into an appropriate animal model with the objective to perform a systematic evaluation of the experimental variables can greatly benefit the field. Furthermore, performing clinical studies in large animals, which show pain conditions that are closer to humans in etiology, can provide additional value. For example, in a placebo-controlled, randomized, double-blind clinical study, intra-articular injection of BoNT/A reduced the joint pain in osteoarthritic dogs [63]. In another randomized, placebo-controlled study in dogs, sub-cutaneous administration of BoNT into the mammary glands 24 h before bilateral, radical, cancer-related mastectomy significantly decreased the post-operative need for rescue morphine analgesia [64]. These findings are well aligned with those showing BoNT/A-mediated pain relief in cancer patients after surgery and/or radiation [65].

Overall, while the diverse pool of clinical studies assessing the efficacy of BoNT in pain is an important source of potential new indications, the questions of where, when, and how to inject require systematic assessment in pre-clinical studies. In addition, pre-clinical studies have been essential in building our understanding of potential mechanisms of BoNT-induced pain relief [28,66,67]. Testing BoNT in disease-relevant animal models, including those in rodents and in larger species, combined with a better understanding of the mechanisms that are involved, can build a solid case for initiation of clinical studies in a new pain or headache indication.

### 3.2. Osteoarthritis

Osteoarthritis (OA) is the most common form of arthritis in humans and OA-related joint pain is a major health concern [68]. Since no disease modifying agents for OA exist, the clinical focus is on pain management and minimizing the functional impairment of the joint.

In OA, changes to the joint articular cartilage surfaces and underlying cartilage matrix lead to the loss of joint space and joint misalignment, as well as inflammation of the joint synovium [69,70]. Fissures and fractures in the cartilage surface appear. Changes at the interface between cartilage and bone, the osteochondral junction, allow for the penetration of sensory and sympathetic nerves from the richly innervated bone marrow [70]. The inflammation eventually causes both the peripheral and central sensitization of neurons, leading to spontaneous joint pain at rest and hyperalgesia. Despite progress on the exact mechanism leading to the pain sensation [71], our detailed mechanistic understanding remains sketchy. However, it seems reasonable to assume that release of sensory neuropeptides such as substance P, calcitonin gene-related peptide (CGRP), and neurokinin A, contributes to the pain sensation in OA [62,72]. Since BoNTs can inhibit the release of these peptides, intra-articular BoNT administration may be able to directly reduce peripheral sensitization, and indirectly reduce central sensitization.

From clinical studies, there is however limited evidence that intra-articular BoNT injections have beneficial effects. Hsieh and colleagues [73] studied 46 patients with symptomatic OA knee who were randomly assigned to a BoNT/A treatment group (100 U Botox^®^ into the affected knee) or a control group. The pain visual analogue scale score in the treatment group significantly decreased from the pretreatment value early after treatment (one week) and was still decreased at the six-month post-treatment follow-up, resulting in a significant difference to the control group at both timepoints. Similar findings were obtained with two other evaluation scales, thus showing that intra-articular injection of BoNT/A provided pain relief and improved functional abilities for the patients with OA knee in this study. However, in a recent double-blind, randomized, placebo-controlled, 12-week trial using a single ultrasound-guided intra-articular injection of BoNT/A in 121 patients no significant differences in clinical efficacy parameters were found between Botox^®^ and placebo in the entire population [74]. In another study recently completed in 176 patients with OA knee with Botox^®^, no statistically significant difference was observed on any of the parameter assessing pain between the onabotulinum toxin and placebo (NCT02230956). Both of the studies add to earlier clinical studies using intra-articular BoNT injections in various rheumatic conditions that were recently reviewed by Khenioui and colleagues [62]. Although the 16 reviewed studies were heterogeneous and had various shortcomings that prohibited the reaching of a generalized conclusion with a satisfactory level of confidence, the reviewing authors did notice that they provided some trend towards the anti-nociceptive effect of intra-articular BoNT/A. Such results show that more research is necessary to understand the mechanism of action and the behaviour of the botulinum toxin when being injected intra-articular. Furthermore, the potential analgesic effects of intra-articular BoNT injections in clinical studies could be explained based on the results of preclinical studies [63,75,76,77,78]. New botulinum toxins that are engineered to specifically target some pain receptors and act on some biomarkers could be developed in future as novel therapeutics.

### 3.3. Overactive Bladder and Neurogenic Detrusor Overactivity

The bladder is a densely innervated organ, and its functions (storing and discarding urine) are under the synergistic control of the sensory, parasympathetic, and somatic nervous system [79]. Overactive bladder (OAB) is a syndrome where urinary urgency is often accompanied by frequency and nocturia. The mechanisms underlying OAB are still a matter of debate. A ‘myogenic’ hypothesis proposes that OAB results primarily from detrusor myocyte overexcitability, whereas a more recent, ‘urothelium-based’, hypothesis highlights the action of the mucosa on afferences sensitization and overactivity. Interestingly, specific cell types, such as urothelial cells [80], and, more recently, telocytes [81], have emerged as pivotal in the development of pathological conditions. Neurogenic detrusor overactivity (NDO) is described in patients with neurologic lesions (e.g., multiple sclerosis, spinal cord injury) where a sacral spinal micturition reflex develops through the activation and the remodelling of C fibres.

Oral antimuscarinic agents are the first line pharmacological therapy for urine storage dysfunction. BoNT started being used off-label in the early 2000s as a second line therapy in OAB [82]. Soon, it became clear that BoNTs action was not limited to its action on parasympathetic structures and acetylcholine release controlling detrusor contractility, but that it was also targeting sensory afferents. Animal models showed that BoNT/A was able to decrease the release of ATP and increase the level of NO [83,84], thus reducing the bladder afferences sensitization, and also decrease CGRP and substance P release, acting on the inflammatory component that may accumulate in bladder dysfunction [85]. Furthermore, clinical evidence highlighted the effect of BoNT/A on nerve growth factor (NGF) release, as well as TRPV1 expression, which are involved in the development of detrusor overactivity [86,87].

**Figure 1:**
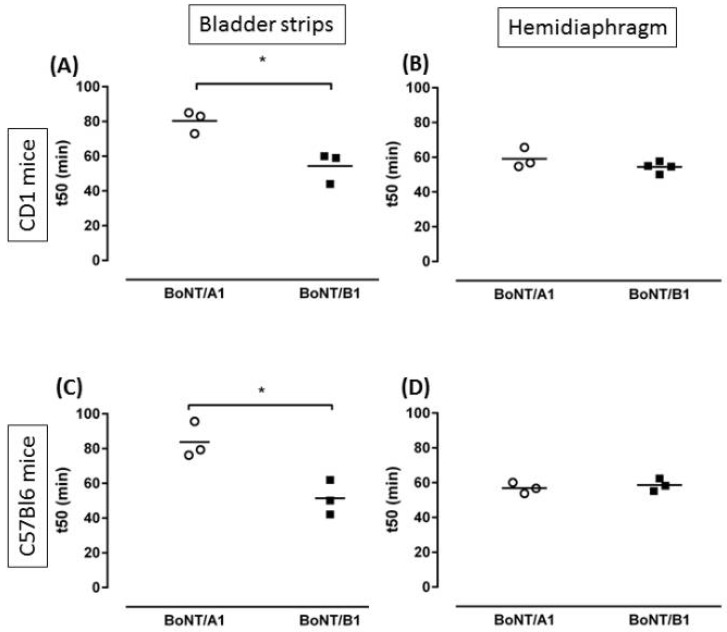
Whatever the mouse strain, BoNT/B1 is significantly more potent than BoNT/A1 in a model of detrusor contractions (**A**,**C**), while the potencies of BoNT/A1 and BoNT/B1 were equal in the phrenic nerve-hemidiaphragm preparation (**B**,**D**).* *p* < 0.05 (unpaired Student’s *t* test) [89].

Moreover, clinical data suggest that BoNT/B may be efficacious at lower doses in the autonomic nervous system of the human bladder when compared to the somatic nervous system, and this was also shown by experiments in rodents [88,89], see also Figure 1. Therefore, there may be a therapeutic opportunity for treating patients with different serotypes [3], within the limits of species sensitivity, as discussed earlier, in Section 2. SV2/SNAP25 are more abundant in cholinergic as compared to sensory fibres in the human bladder [90], which may explain some cases of urinary retention in patients being treated by intradetrusor injections of BoNT/A. Intrathecal administration of BoNT/A in spinal cord-injured rats, the prototypical animal model of neurogenic detrusor overactivity, resulted in normalization of pathological bladder contractions and bladder basal pressure [91]. This may open research opportunities for natural or engineered neurotoxins that are targeting the afferent and pivotal component of the disease with a different therapeutic profile compared to BoNT/A.

### 3.4. Wound Healing

The healing of a wound comprises four overlapping phases: haemostasis, inflammation, tissue proliferation, and remodeling (for review see [92]). Increased metabolic activity and inflammation during healing can induce muscle contractions around the edges of the wound, which in turn, creates repetitive tension on the wound [93]. This has the potential to not only delay the healing of the wound, but also to increase fibrosis and induce or aggravate hypertrophic scarring. Chemoimmobilisation of the musculature adjacent and under a surgical wound through injection of BoNT could thus be beneficial for the overall wound healing process and the cosmetic appearance of the healed wound.

Indeed, there is clinical evidence that BoNT/A may have beneficial effects in wound healing. For example, using a visual analogue scale in patients who had undergone surgery for facial wounds, Ziade and colleagues [94] found a statistically significant improvement in scarring of the tissue in those patients who had been injected with BoNT within three days post-surgery, compared to patients not injected with BoNT. However, no statistically significant differences were found between the two groups in this study when using other assessment methods. Similarly, a systematic review of the literature for the use of BoNTs for the prevention of hypertrophic scars that included ten clinical studies, found improved cosmetic outcomes among certain studies. However, due to the heterogeneity of the studies, as well as other factors, the authors concluded that the data were not supportive of the clinical usage of BoNT for this indication at this time, but recommended randomized controlled trials to reach a firm conclusion [95]. At the time of writing, April 2018, two randomized controlled clinical trials are currently ongoing (NCT02623829 and NCT02886988).

While the clinical data are thus suggestive, but presently are not yet conclusive, preclinical experimental work has provided evidence that BoNTs do have beneficial effects in wound healing. For example, using a study design that allowed for each animal to be its own control, Lee and colleagues [96] showed significant differences in wound size between BoNT-treated and untreated control wounds. The treated wounds also showed less infiltration of inflammatory cells, a smaller number of fibroblasts, less fibrosis, and a lower expression of transforming growth factor (TGF)-β1, as compared to the control wounds. TGF-β1 is a fibrotic cytokine that has pleiotropic actions in wound healing [97], and it is involved in the formation of hypertrophic scars. The finding by Lee and colleagues [96] that BoNT-treated wounds show a lower expression of TGF-β1 may be the result of the chemoimmobilisation of the muscle. However, BoNTs may also directly interfere with the expression of TGF-β1 in fibroblasts and fibroblast proliferation [98,99].

## 4. BoNT Effects Remote from the Site of Injection

### 4.1. Neuronal Retrograde Transport and Central Effects

The vast majority of clinical effects that are exhibited by BoNTs are attributed to their well-established actions at the site of injection [100,101,102,103,104,105]. However, in some instance, the in situ activity of BoNT seems insufficient to fully explain its clinical efficacy, thus raising the idea that other “non-classical” actions on the central nervous system might also be involved [51,106,107]. Indeed, some data have shown that peripherally injected BoNT triggers distant changes at various central levels.

For example, the involvement of cortical networks after BoNT/A administration has been reported. Patients suffering from cervical dystonia were shown to display a higher neuronal excitability (measurement of P22/N30 cortical component of median nerve somatosensory evoked potentials) and reduced gray matter volume that was assessed by magnetic resonance imaging (MRI) in discrete cortex areas [108,109]. Local injections of BoNT/A in cervical muscles had long-lasting beneficial effects on these central alterations.

Some studies also assessed the effect of BoNT/A peripheral muscular injections on brainstem activity in patients with blepharospasm [110] or dysphonia [111]. In the first study, unilateral BoNT/A injections in the *orbicularis oculi* decreased in a strong and similar fashion the blink reflex after the stimulation of supra-orbital nerve in both ipsilateral and contralateral muscles. On the other hand, the activity of injected muscle measured by electromyography was not inhibited in the same proportion as the latter parameter. According to the authors, the beneficial action of BoNT/A in blepharospasm is thus more likely due to changes in brainstem interneuronal pathways than local muscular action [110]. The same conclusions were reached by Bielamowicz and Ludlow [111], who observed after unilateral BoNT/A administration in *thyroaritenoid* muscles decreased activation levels and spasmodic bursts that were measured by electromyography in both injected and contralateral muscles.

Central changes at the spinal cord level were also demonstrated. Marchand-Pauvert, Aymard [112] investigated, in patients exhibiting lower limb spasticity, the recurrent inhibition from a BoNT/A-injected muscle (*soleus*) to a distant untreated muscle (*quadriceps femoris*). After stimulation of the tibialis nerve that contains motoneurons innervating the *soleus*, the recurrent inhibition was found to be decreased in comparison with measurements that were recorded before treatment. The authors hypothesized that this effect was unlikely due to the local action of the neurotoxin in the injected muscle or on spindle afferent input, but is more likely due to a modification of spinal synaptic transmission. Indeed, they suggested that BoNT/A underwent retrograde neuronal transport from the injected muscle to the spinal cord and locally reduced the stimulation of motoneuron terminals on Renshaw cells, inhibitory interneurons projecting to motoneurons innervating the *quadriceps* muscle. This probably led to the decreased recurrent inhibition of the latter muscle [112]. A similar study obtained comparable findings [113], providing more evidence for a central action of BoNT in humans as a result of a putative neuronal retrograde transport.

Cellular and electrophysiological alterations in Renshaw cells were also found in rats that were injected peripherally with neurotoxins [114,115], indicating that such a phenomenon could also exist in animals. Antonucci, Rossi [21] demonstrated that BoNT/A injections in the hippocampus, whisker pads, or optic tectum led to the appearance of cleaved SNAP25 in the contralateral hippocampus, in the neuropil surrounding facial neurons soma, and in the retina, respectively. Interestingly, in the latter pathway, pretreatment with colchicine (which inhibits microtubule-dependent retrograde transport) abolished the appearance of cleaved SNAP25 in the retina. These first sets of data suggest that either an active form of BoNT/A or cleaved SNAP25 itself could have been transported from nerve terminals to neuron soma. The authors tried to answer that question by injecting BoNT/A in the optic tectum of rats, and, three days later, concomitantly sectioning the optic nerve and injecting BoNT/E in the eye vitreous. As expected, BoNT/E rapidly eliminated SNAP25 cleaved by BoNT/A from the eye. However, 25 days later, the appearance of SNAP25 cleaved by BoNT/A was observed, suggesting the presence of active BoNT/A in the same region [21].

**Figure 2:**
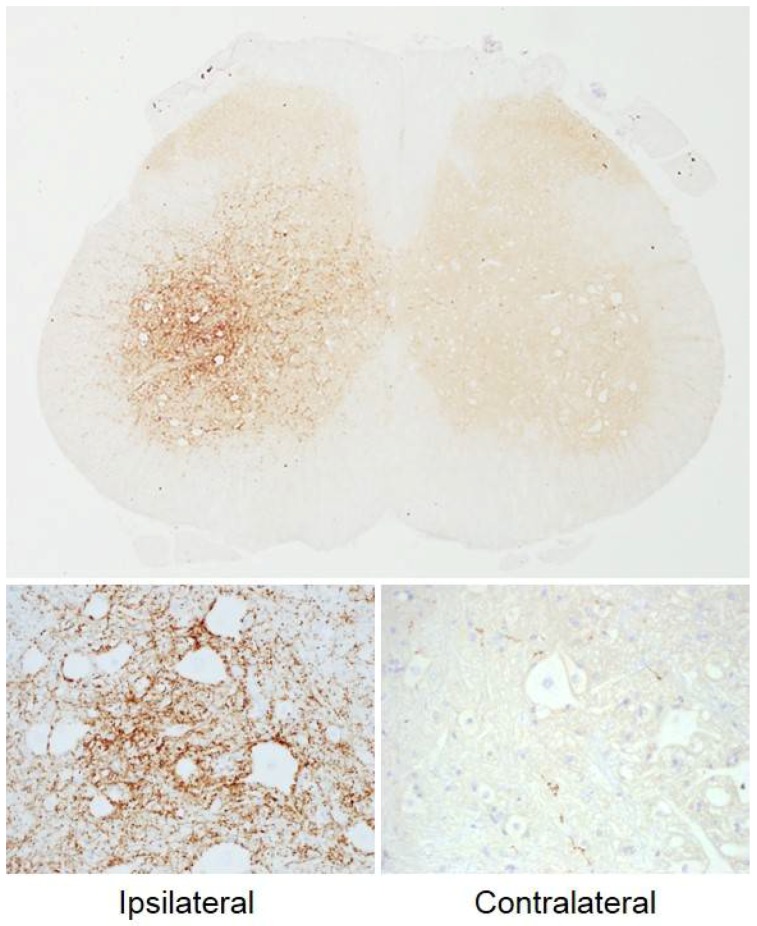
Immunohistochemistry staining of cleaved SNAP25 in rat lumbar spinal cord after BoNT/A injection in the left gastrocnemius muscle. Intense staining was found in the ipsilateral ventral horn neuropil, and only traces were found in the contralateral side (SL, VM, unpublished data).

Similar results were found in various in vivo studies, with the appearance of cleaved SNAP25 at the spinal cord level following peripheral BoNT/A injections in rat or mice hindlimb (Figure 2) [52,116,117,118], and in the brainstem after BoNT/A administration in the whisker pad [119,120,121] or in the optic tectum [122]. However, direct evidence for a retrograde transport of BoNT would be to detect an active form (either holotoxin or light chain) in central regions after peripheral administration. To our knowledge, this has never been demonstrated, although a work from Wang, Martin [123] showed the presence of fluorescently tagged BoNT/A heavy chain (thus not active) in mice spinal cord after hindlimb injection.

Data from in vitro experiments shed some light on this question. Indeed, numerous works were able to demonstrate, in motoneuron or hippocampal neuron cultures, retrograde transport of either BoNT/A holotoxin or its heavy chain domain [49,120,123,124,125]. Interesting to note, heavy chains of both BoNT/A and tetanus toxin were found to share the same retrograde carriers when being retrogradely transported [120]. More precisely, BoNT/A heavy chain is thought to be internalized in nerve terminals within a specific pool of non-recycling synaptic vesicles [124], and undergo axonal retrograde transport in autophagosomes that finally fuse to lysosomes at the soma level [123]. In an elegant study, Bomba-Warczak, Vevea [49] used microfluidic chambers to demonstrate that application at the soma side of anti-BoNT/A antibodies or heavy chain competing with holotoxin prevents the appearance of cleaved SNAP25 in this compartment following BoNT/A application at the axon side. These data are thus in favor of a retrograde transport of BoNT/A holotoxin to the soma of the primary neuron, which is then exposed to the extracellular medium before entering the secondary neuron, leading to distal effects [49].

In vitro data are thus suggestive of retrograde transport of BoNT/A besides its classical mechanism of action, underlining some clinical observations. However, the axonal transport of a functional form of the toxin still needs to be clearly demonstrated in vivo. Apart from BoNT/A, little is known about the potential retrograde transport of other BoNT serotypes. Some data support the existence of such phenomenon for BoNT/E, but apparently, to a lesser extent than for BoNT/A [21,120,125]. BoNT/B [126] and BoNT/D [49] were also shown to undergo retrograde transport.

### 4.2. Diffusion within the Injected Muscle, Local Spread and Contributing Factors

When injected in a tissue, BoNTs present with a remarkable safety profile, mostly remaining within the injected organ, allowing for their use for various medical applications, as indicated in various reviews and meta-analyses [127,128,129]. While the diffusion of the injected toxin is somehow expected within the injected muscle or in very close neighboring muscles, local spread can be observed beyond the site of injection leading to different unexpected effects, depending of the targeted area. Local adverse effects that are associated with the intramuscular use of BoNT are thus due to excessive local muscle weakness and leakage to nearby muscles, and are reported as leading potentially to severe adverse events [130]. When used for the treatment of blepharospasm the most frequently reported adverse reactions were eyelid ptosis (21%), superficial punctate keratitis (6%), and dry eye (6%) for Botox^®^ and eyelid ptosis (19%), dry eye (16%), and dry mouth (16%) for Xeomin^®^ (for further details see prescribing information for both products, FDA). When used for the treatment of cervical dystonia, the most frequently reported adverse reactions were dysphagia (19%), upper respiratory infection (12%), neck pain (11%), and headache (11%) for Botox^®^; muscular weakness (16%), dysphagia (15%), dry mouth (13%), and injection site discomfort (13%) for Dysport^®^; and, dysphagia (18%), neck pain (15%), and muscle weakness (11%) for Xeomin^®^ (for further details see prescribing information, FDA). Theses adverse effects suggest that unbound toxin moves away from the muscle, most likely through the extracellular space and determined by a concentration gradient. The dose, volume, and injection techniques that are used to target the different muscles are important to limit the occurrence of such adverse reactions.

**Figure 3:**
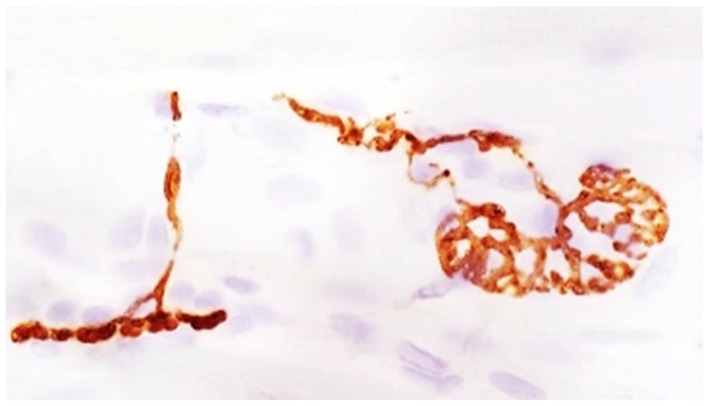
Immunohistochemistry staining of cleaved SNAP25 in rat muscle injected with BoNT/A (SL, VM, unpublished data).

The evaluation of local spread has been conducted in humans and animal models using electrophysiology and/or tissue markers, such as cleaved SNAP25 or other surrogate markers (for example, see Figure 3). Using electrophysiology and periodic acid-Schiff stain to identify glycogen content in muscle fibers, it was estimated that BoNT/A can pass through the *tibialis* muscle fascia (thick fibrous tissue covering the muscle), and the presence of the *tibialis* fascia can reduce the local spread by 20% in the rat [131]. This local spread was also confirmed in larger species, such as in the cat [132], and primates [133]. In rabbits that were injected with 2.5 U/kg of BoNT/A (unknown origin) by i.m. route in the *longissimus dorsalis* muscle, the intra-muscular diffusion of the toxin from the injection site was reported over a distance of 3 to 4.5 cm, and local spread across facial planes identified over a distance of 1.5 to 2.5 cm with the same intensity as within the injected muscle [134]. In the mouse, N-CAM (a marker that is expressed in denervated muscles) was strongly expressed in the myofiber of the injected *tibialis* muscle, but limited expression was detected in the ipsilateral *gastrocnemius* and *quadriceps femoris* muscles of the injected leg, suggesting a limited local spread in this model using three different BoNT/A formulations (Dysport^®^, Botox^®^, and Xeomin^®^) [135]. 

Regarding the effect of injection volume, little is known and the literature does not support a substantial influence on local spread. In humans treated for dynamic forehead lines at relatively low doses of BoNT/A (5 U Botox^®^ /injection site) the area affected was slightly greater with an injected volume of 250 µL when compared to a volume of 50 µL; the increase in local diffusion was estimated to be around 1.5 times higher (+50%) for a five times larger injection volume [136]. Similarly, in humans, doubling the concentration (same dose in half the injection volume) resulted in significantly greater effects on the injected muscle, as assessed by CMAP amplitude evaluation (effect on the intra-muscular diffusion). However, effects in nearby muscles were only slightly dependent on the injected volume, with limited differences between the groups [137].

### 4.3. Systemic Spread and Contributing Factors

Beyond local spread, leakage from the injection site may occur, resulting in a hematogenous spread and systemic exposure, leading to symptoms that are consistent with the mechanism of action of botulinum toxin [138]. Hematogenous spread may result in signs of asthenia, generalized muscle weakness, diplopia, blurred vision, ptosis, dysphagia, dysphonia, dysarthria, urinary incontinence, and breathing difficulties. These symptoms have been reported hours to weeks after injection. Extremely rare cases of swallowing and breathing difficulties have been reported [138,139,140], as indicated in all the commercially available BoNT labels. These observations are extremely rare and BoNTs present with a very good benefit/risk balance [128,129].

Various factors may affect the fate the injected toxin and its effect in the body, such as volume/concentration, amount of toxin, formulation, needle size, number of injections, precision of injection, and the biologic properties of the injected toxin (mechanism of action, duration of action, half-life in tissues), as well as the anatomy of the target area and tissue type [138,141].

BoNT serotype is also an important contributing factor affecting systemic spread. There was potentially greater spread to nearby and remote non-injected muscles that are associated with BoNT/A when compared to BoNT/B at equivalent efficacious doses in the mouse and primate using the CMAP test [134]. Similar observations were reported in the mouse, in which i.m. LD_50_ was 13.9 U/kg for BoNT/A (Botox^®^) and 104.6 U/kg for BoNT/B (Myobloc^®^), while safety margins (LD_50_/ED_50_) were lower for BoNT/B (5.4) than BoNT/A (13.9) [142], suggesting that BoNT/B is less safe. Other factors probably influence spread and its clinical consequences (e.g., toxin half-life in tissues and blood, distribution of receptors, co-receptors, and SNARE proteins in the targeted physiological system [muscular or autonomic], etc.). This was confirmed by a Japanese group using the CMAP test in the injected and contralateral limb muscle in the rat to compare toxins at equivalent efficacious doses [143]. They determined that BoNT/CD is the most prone to spread remotely, and that BoNT/D is least prone to spread. The rank order of BoNT serotypes based upon spread in this study in rats was CD > A > E > C > B > F > D. Interestingly, when ranked based upon safety index a different order was obtained: F > C > D > E > A > CD > B, with BoNT/F being the safest. Recent data obtained using CMAP amplitude and/or cleaved SNAP25 immunohistochemistry in remote muscles, also support the dose dependent systemic spread effect in rats that were injected with BoNT/A [116,117].

### 4.4. New BoNT Therapeutics with Decreased Spread

Spread could be an important differentiating factor in the BoNT therapeutic landscape. BoNT/A3 [144] and BoNT/A2 [38] are two subtypes that may offer reduced spread. BoNT/A2 has been reported as showing less spread to neighbouring muscles than BoNT/A1 [38]. In a study with healthy volunteers, the effect of 6.5 U BoNT/A2 and 10 U BoNT/A1 that is injected into the *extensor digitorum brevis* muscle on either side of the same subject were compared [37]. Although a similar onset, duration, and magnitude of the effect was seen for the two toxins, there was less spread of the effect to the neighbouring *abductor hallucis* muscle for BoNT/A2 compared to BoNT/A1. In a randomized double-blinded controlled phase 2/3 study in post-stroke spasticity, hand grip strength on the unaffected side was significantly reduced after BoNT/A1 treatment, but not after BoNT/A2, which is again consistent with less spread of A2 when compared to A1. In a later study, BoNT/A2 was reported to have reduced systemic toxicity, which is in agreement with the reduced spread that was reported in the clinical studies above [145], and also reduced anterograde transport compared to BoNT/A1, as measured by muscle contraction and immunocytochemical analysis [31,117]. However, despite these encouraging results, there is much lower number of safety data in humans for BoNT/A2 as compared to BoNT/A1, and as such, more human exposure to BoNT/A2 will be needed prior to establishing BoNT/A2 as a differentiated therapeutic. The biological properties and potential clinical utility of BoNT/A2 are discussed more fully in Section 2 above.

## 5. Novel Formulations

### 5.1. Current Products and Challenges Associated with Formulation of BoNTs

The BoNT formulations for the main commercially available products have existed for over 20 years. The formulation of BoNTs is challenging, owing to their structural complexities, the analytical tools that are available, and the low product concentrations used. The extremely high potency and selectivity of BoNT for cholinergic neurons, with a human lethal dose of parenteral injection estimated as between 0.1–1 ng/kg [146], requires that pharmaceutical preparations of BoNT comprise vanishingly small quantities of the toxin itself. Drug products have been shown to contain between 0.44 and 3.24 ng of the 150 kDa toxin per vial [8], and as such, present unique challenges for the formulation and the analytical strategy employed to assess stability. BoNTs in this respect differ from conventional biologics where much of the focus of formulation is on controlling protein aggregation [147]. One of the primary challenges in the formulation of BoNTs is minimising loss during manufacture and storage. BoNTs are also very susceptible to denaturation due to surface denaturation, heat, and alkaline conditions. Due to these contributing factors, the lyophilisation or freeze-drying of the toxin complex or the neurotoxin is the favoured method of distributing the product in a form that is stable and readily used by the clinician [9]. All BoNT/A clinical products currently available, apart from in Japan, Korea and Taipei, are dried powder products that need to be reconstituted for use. The BoNT/B product has been provided as a liquid, which is ready for use, since its license approval in December 2000. This product may be painful when injected, however, due to the acidic pH of the solution. Medytox was the first BoNT/A liquid product launched, Innotox^®^, but limited to Korea, Taipei, and Japan, as of today. Allergan licenced worldwide rights to Innotox outside Korea from Medytox. Most of the major clinical BoNT/A manufacturers are working to develop liquid formulations. Liquid formulation is considered the next generation of toxin products because of its convenience and ease of administration. Recently, Ipsen has reported a phase 2 clinical trial of a ready-to-use liquid formulation of abobotulinumtoxinA (abobotulinumtoxinA solution for injection, ASI). This formulation was shown to be efficacious, with comparable results to reconstituted abobotulinumtoxinA, and to have a favorable safety profile in subjects with severe to moderate glabellar lines [148]. Another feature of all the existing product formulations is the presence of human serum albumin (HSA) as a stabilising agent. This is not desirable due to the theoretical risk of contamination with pathogens. As part of the development of next generation liquid formulations, companies are exploring formulations that have different stabilisers and components, including the omission of excipients from animal origin and HSA.

### 5.2. Slow-Release Formulations

Advanced formulations for BoNT include the slow or sustained release formulations, which aim to release BoNTs over a defined period in a controlled manner. This may be through colloidal carrier technology and/or exploiting the thermo-responsive nature of polymeric solutions. Depending on the clinical application, such formulations may provide a benefit to the existing delivery methods that are used. The clinical use of a slow-release formulation or depot requires careful consideration. In many indications, the accuracy of injection is a critical factor during treatment, with skilled injectors depositing the BoNT at the neuromuscular junctions to minimise the spread to adjacent muscles. In many treatments, this is performed in multiple locations within a muscle to achieve the desired outcome. A successful slow-release depot would, therefore, have to remain in situ without migration for the period of release. The duration of BoNT therapeutic benefit in movement disorders is typically 3–4 months [149]. Currently, the sustained release profile for most clinically applicable depot formulations is no more than approximately seven days, which would not produce a significant improvement in duration for BoNTs. For a significant improvement in the duration of BoNT effects, release over 3–4 weeks is likely to be necessary. The challenge of formulating to achieve the desired release profile is therefore much greater and further compounded by the additional complexity of needing to stabilise a large and complex protein at 37 °C for this duration. Also, any depot would need to be sufficiently small that the lasting presence is not uncomfortable for the patient.

### 5.3. Analytical Challenges

If a slow-release formulation were to be developed, it would be desirable to demonstrate the release profile of the drug over time, both in vitro and in vivo. The concentration at the point of administration, however, is already in the picomolar range, a level at which most pharmacokinetic studies are performed. It seems likely that the released material from a slow-release depot would be at even lower concentration, and over a greater time. Accurate determination of this profile is important for a product possessing such high potency as BoNT [146]. This increases the analytical challenge in preparing such a formulation, ensuring the accuracy of the final dose, and developing a consistent release profile. The absence of circulating biomarkers for the intoxication of neurons by the toxin further increases the complexity, as the pharmacological measure is only obtained from measurement of the local paralysis, electrophysiology, or other functional responses. With recent advances in analytical sensitivities [150,151], the accurate detection of BoNT at the required concentrations is beginning to become achievable, but the technologies are still not in routine use. This analytical improvement will be vital for development of slow-release formulations of BoNTs.

## 6. Novel Delivery Methods

Currently clinical BoNT products are delivered by local injection into the target tissue. This can be a painful process and requires clinical expertise on the part of the administrating physician to ensure the correct local delivery and avoid the application to adjacent tissues resulting in a lack of therapeutic efficacy and/or off-target effects. For conditions such as hyperhidrosis, application can require multiple subcutaneous injections in sensitive locations, such as the palm or axilla, resulting in discomfort or even pain for the patient. Injection in such situations can require local analgesia or pain block. In bladder applications, where multiple injections via cystoscopy are required, this can require local or even general anaesthesia. Other limitations to the injection of BoNT products include erythema, swelling, and potential for infection. In aesthetic use in facial areas, there is also the risk of bruising and/or bleeding. Bleeding can also be an issue for injections of BoNT into the bladder urothelium. Development of needle-free delivery would therefore be of great benefit in such circumstances, requiring less specialised clinician input, increased convenience, and reduced discomfort/pain for the patient. Non-invasive delivery of BoNTs is challenging owing to the biological barriers limiting successful access to the intended tissue and the large size of the protein. Approaches under development involve either a physical or chemical approach to transiently permeabilise the barrier and enable entry of the BoNT. To date, all of the needle-free approaches in development are aimed at the relatively superficial delivery and not replacing intramuscular injection to deeper muscles involved in many of the movement disorders where BoNT is used therapeutically.

### 6.1. Transdermal Delivery

Development of transdermal drug delivery methods has a long history and has already made a significant contribution to medical practice [152], although largely for small molecule drugs to date. A number of investigators have studied the ability of enhancers to increase skin permeability and enable the transdermal entry of BoNT. One approach that has received a lot of attention is the use of Cell Penetrating Peptides (CPP), and particularly ones that are derived from the transactivator of transcription (TAT) protein of human immunodeficiency virus. Revance Therapeutics Inc. have progressed this approach for transdermal delivery of BoNT/A to the clinic for both lateral canthal lines and axillary hyperhidrosis. The neurotoxin construct that was progressed by Revance, RT001, consists of a purified 150 kDa BoNT/A protein formulated in a poloxamer gel containing a CPP, RTP004, a single chain peptide consisting of 35 L-amino acids. RTP004 has two domains, a core sequence of 15 lysines that confer positive charge to the peptide, and a protein transduction domain that is derived from residues 49–57 of the TAT protein [153]. The CPP associates with the neurotoxin protein non-covalently via ionic interactions between cationic regions within the CPP and the negatively charged surface of the neurotoxin protein. The resulting complex enables transcutaneous flux of the neurotoxin via cell to cell micropinocytosis. Varying the CPP was found to vary the delivery characteristics of the payload, enabling the delivery to different depths of penetration; the longer the peptide the deeper the penetration. In RT001, the CPP is formulated to deliver the BoNT/A to the mid-dermis appropriate for treatment of lateral canthal lines and hyperhidrosis. Phase 1 studies with RT001 showed it to be safe and tolerable [154]. Subsequently, a number of phase 2 studies demonstrated RT001 to be safe and effective in the treatment of moderate to severe lateral canthal lines or hyperhidrosis [155,156,157]. While in 2015, Revance announced that it had commenced dosing patients in a Phase 3 pivotal study to evaluate the safety and efficacy of RT001 for the treatment of lateral canthal lines the study did not achieve its co-primary endpoints, and was discontinued. Revance is now focused on developing an injectable form of BoNT/A combined with its proprietary peptide technology, RT002, which is claimed to spread less from the site of injection and to give a greater safety margin and enable the enhanced duration of therapeutic benefit.

Another approach to transdermal delivery of BoNT/A, which was developed by Anterios Inc., was the use of liposomes of a controlled size range to deliver BoNT/A for hyperhidrosis. Anterios was acquired by Allergan plc in 2016. In 2015, the lead Anterios formulation, ANT-1207, entered a phase 2 trial (NCT02479139) for axillary hyperhidrosis. Allergan have yet to report on the outcome of this study.

Chajchir and colleagues reported on the use of a microcrystal and nanoparticle formulation, InParT (Transdermal Corp), reconstituted with Botox^®^ at 2 U/mL for transdermal delivery in the treatment of upper facial lines [158]. The formulation was found to be effective versus placebo based on patient reported Facial Line Outcome scores. The formulation was applied daily for a period of 4 to 7 weeks, with a 12 week follow up period. The improved outcome scores were maintained during the whole study period. Given the very low dose of BoNT/A that was employed, this result is surprising, but may reflect the frequent initial dosing regimen.

Transdermal delivery of BoNT/A using a short synthetic peptide, TD-1, as an enhancing agent has been reported to reduce plasma extravasation and blood flow changes that are evoked by electrical stimulation of the hind-paw in a rat model of neurogenic inflammation [159]. Peptide-mediated transdermal delivery of BoNT/A was proposed to offer an easy and non-invasive route of administration for the treatment of neurogenic inflammation.

Perhaps the most unusual and potentially risky uses of CPP to achieve transdermal delivery of BoNT was the direct fusion of the TAT peptide to the light chain of BoNT/A [160,161]. This was reported to enable the direct application and delivery of the light chain into cells without the need for any of the other domains of BoNT that mediate neuronal specific binding and the translocation of the light chain into the cytosol [160]. Transcutaneous delivery through mouse skin was also evaluated by immunohistochemistry. The authors discuss the therapeutic potential of such an approach, but it did not consider the lack of selective targeting or the potential for non-specific entry into any cell that was exposed to the fusion protein. Such a lack of cell selectivity must significantly increase the risk of non-specific toxicity and represent a significant safety barrier to therapeutic application of such a fusion protein.

The requirement for an enhancer to enable the transdermal delivery of BoNT is demonstrated by the inability of BoNT/A applied directly to the skin to impact palmar hyperhidrosis [162]. Without any enhancer to allow for the entry of the neurotoxin across the dermal barrier no effect above control was observed, clearly showing the inability of a large multi-domain protein, like BoNT, to cross the dermal layer unaided.

In addition to chemical enhancing agents, the use of physical approaches to disrupt the permeability barrier of the skin and enable entry of BoNT has been explored. Kavanagh and Shams reported the use of iontophoresis to deliver BoNT/A for the treatment of palmar hyperhidrosis, with an improvement in the sweat rate of 66%, which is a similar result to that obtained with intradermal BoNT/A [163]. Subsequently, Pacini and colleagues confirmed in rats that pulsed current iontophoresis delivered BoNT/A through living skin [164]. Following iontophoresis, BoNT/A was clearly detected in association with cutaneous striated skeletal muscle fibres in the deep dermis of the rat skin. Transdermal delivery by jet nebulization of BoNT/A in combination with lidocaine has been reported to be an effective and painless method for the successful treatment of palmar, plantar, and axillary hyperhidrosis [165]. An emerging technology for transdermal drug delivery is the use of microneedles [166], and the use of microneedles for the delivery of BoNT/A into the skin has been reported [167]. As yet, however, there are surprisingly few publications on this route of transdermal delivery for neurotoxin.

### 6.2. Transurothelial Delivery

The other major application of BoNT to have received considerable attention in regards to needle-free delivery is the use in the bladder for treatment of OAB and NDO. The current procedure is invasive, requiring cystoscopic guidance and multiple injections into the bladder, and often necessitating intravenous sedation or anaesthesia. If a less invasive, needle-free method of administration could be demonstrated to be effective, this would be preferable. There is also evidence that whilst injection of BoNT into the bladder wall impacts both the afferent and efferent nerves, approaches that avoid injection impact only afferent nerves, which may be therapeutically advantageous [168].

Intraluminal instillation of BoNT/A without injection failed to deliver toxin into the bladder wall due to the impermeability of the bladder urothelium to large macromolecules and degradation of the toxin by urine proteases [169,170]. In order to enable the entry of BoNT into the bladder wall, it is necessary to use chemical agents to denude the urothelium. One such approach is the use of the organic solvent dimethyl sulphoxide (DMSO) [170]. The safety and the efficacy of co-administration of BoNT/A with DMSO has been demonstrated in a clinical trial in women with refractory OAB [171]. Protamine sulphate has also been employed to denude the bladder urothelium and aid access of BoNT/A to the bladder wall [169,172]. Protamine sulphate pre-treatment of bladder prior to the instillation of BoNT/A has been reported to be safe and potentially effective in patients with refractory OAB [173]. Instillation of BoNT/A was reported to reduce the inflammatory effects that are caused by the prior injection of cyclophosphamide in a rat model of cystitis [174]. In this case, it was proposed that the permeability barrier of the bladder urothelium had been impaired by the cyclophosphamide-induced inflammation enabling access of the instilled BoNT/A. Hyaluronan-phosphatidylethanolamine (HA-PE) has been reported to be another potential chemical agent allowing for bladder instillation of BoNT/A to be effective [175]. Based upon SNAP25 cleavage, the instillation of BoNT/A with HA-PE was as effective as intra-detrusor injection in enabling BoNT/A delivery into the bladder.

Inert heat sensitive hydrogel preparations that increase the residence time of neurotoxin adjacent to the urothelium and allow for slow release exposure has been used to enhance the effectiveness of instilled BoNT/A. OnabotulinumtoxinA embedded in the hydrogel TC-3 has shown efficacy in the treatment of OAB [176]. TC-3 is produced by an Israeli company UroGen Pharma Ltd. (Ra’anana, Israel) as RTGel^TM^. In 2016, UroGen licensed the worldwide rights to develop RTGel^TM^ in combination with BoNT/A to Allergan Inc. (Dublin, Ireland), who are currently conducting phase 2 studies in OAB. Liposomes are being developed as a delivery vehicle enabling intravesical BoNT/A delivery in the bladder by the US biotech Lipella Pharmaceuticals [177]. Bladder instillation of liposome encapsulated BoNT/A was effective at treating the symptoms of OAB in patients [178].

As with transdermal delivery, in the bladder, physical methods to enhance the permeability of the urothelium have been employed as well as chemical. Low energy shock waves have been reported to enable delivery of intravesical BoNT/A into the bladder wall and block acetic acid-induced hyperactivity in a rat model of bladder hyperactivity [179]. The use of intravesical electromotive BoNT/A delivery in the bladder has been widely studied, both in animal models [180] and clinical application [180,181,182,183]. Intravesical electromotive BoNT/A has been reported to be effective in women with OAB [181] and in NDO in children [182,184]. In children, a long-term follow-up study has shown it to be a safe and effective method of treating NDO resulting from myelomeningocele [183].

### 6.3. Transepithelial Delivery

One other area where needle-free delivery of BoNT/A has been explored is topical intranasal delivery. Successful intranasal delivery of BoNT/A and the relief of symptoms of allergic rhinitis in rats was achieved using the proprietary CPP RT001 formulation [185]. Effective relief of idiopathic rhinitis in some patients has been reported following simple intranasal application of a sponge soaked in BoNT/A solution [186].

## 7. Conclusions

BoNTs have proved to be highly effective therapeutic proteins offering symptomatic relief across a wide spectrum of neurological and muscular conditions that are characterized by neuronal hyperactivity. The increasing understanding of the biology of the neurotoxins and the availability of highly differentiated toxin serotypes and subtypes offers the prospect in coming years of expanding this therapeutic benefit and extending it to a greater range of clinical conditions, offering the benefit of being more specific and the efficacy of these unique therapeutic proteins to a much greater number of patients. Whilst botulinum toxins have the advantage of being extremely potent with an extended duration of biological activity, properties that are key to their clinical success, these very characteristics present significant challenges to the scientific field of pharmaceutical development. Furthermore, in the coming years, modified and recombinant toxins would expand even more the future therapeutic utility of BoNTs.

## Figures and Tables

**Table 1:** Characteristics of current major botulinum neurotoxin (BoNT) products.

	AboA ^1^	IncoA ^2^	OnaA ^3^	RimaB ^4^
1st Approval	1991	2005	1989	2000
Serotype	A1	A1	A1	B
Strain	Hall	Hall	Hall	Bean
Purification Method s	Chromatography	Unpublished	Crystallization	Chromatography
Complex Size	>500 kD	150 kD	900 kD	700 kD
Excipients	HSA (125 µg) Lactose	HSA (1 mg) Sucrose	HSA (500 µg) Sodium chloride	HSA (500 µg/mL) Sodium succinate Sodium chloride
Stabilization	Lyophilization	Lyophilization	Vacuum drying	Solution
Solubilization	Normal saline	Normal saline	Normal saline	N/A
pH	~7	~7	~7	5.6
Unitage (U/vial)	300, 500	100, 200	100, 200	2500, 5000, 10,000
Shelf Life (months)	24	36	36	24
Neurotoxin Protein (ng/vial) ^†^	4.35	0.6	5	~25, 50, 100

^†^ Protein (ng/vial) is for entire neurotoxin complex, the total protein load being dominated by albumin. HSA = human serum albumin. ^1^ AboA = abobotulinumtoxinA (Dysport^®^). Dysport^®^ PI, Ipsen, 2015. ^2^ IncoA = incobotulinumtoxinA (Xeomin^®^). Xeomin^®^ PI, Merz, 2015. ^3^ OnaA = onabotulinumtoxinA (Botox^®^). Botox^®^ PI, Allergan, 2015. ^4^ RimaB = rimabotulinumtoxinB (Myobloc^®^/Neurobloc^®^). Myobloc^®^ PI, Worldwide Meds, 2010.

## References

[1] Zhang S., Masuyer G., Zhang J., Shen Y., Lundin D., Henriksson L., Miyashita S.-I., Martínez-Carranza M., Dong M., Stenmark P. (2017). Identification and characterization of a novel botulinum neurotoxin. Nat. Commun..

[2] Hill K.K., Smith T.J. (2012). Genetic diversity within Clostridium botulinum serotypes, botulinum neurotoxin gene clusters and toxin subtypes. Botulinum Neurotoxins.

[3] Peng L., Berntsson R.P.-A., Tepp W.H., Pitkin R.M., Johnson E.A., Stenmark P., Dong M. (2012). Botulinum neurotoxin DC uses synaptotagmin I and II as receptors, and human synaptotagmin II is not an effective receptor for type B, DC and G toxins. J. Cell Sci..

[4] Strotmeier J., Willjes G., Binz T., Rummel A. (2012). Human synaptotagmin-II is not a high affinity receptor for botulinum neurotoxin B and G: Increased therapeutic dosage and immunogenicity. FEBS Lett..

[5] Dressler D., Bigalke H., Benecke R. (2003). Botulinum toxin type B in antibody-induced botulinum toxin type A therapy failure. J. Neurol..

[6] Tao L., Peng L., Berntsson R.P.-A., Liu S.M., Park S., Yu F., Boone C., Palan S., Beard M., Chabrier P.-E. (2017). Engineered botulinum neurotoxin B with improved efficacy for targeting human receptors. Nat. Commun..

[7] Brin M.F., James C., Maltman J. (2014). Botulinum toxin type A products are not interchangeable: A review of the evidence. Biol. Targets Ther..

[8] Frevert J. (2010). Content of botulinum neurotoxin in botox^®^/vistabel^®^, dysport^®^/azzalure^®^, and xeomin^®^/bocouture^®^. Drugs R D.

[9] Pickett A., Foster K. (2014). Botulinum toxin as a clinical product: Manufacture and pharmacology. Clinical Applications of Botulinum Neurotoxin.

[10] Ludlow C., Hallett M., Rhew K., Cole R., Shimizu T., Bagley J., Schulz G., Yin S., Koda J. (1992). Therapeutic use of type F botulinum toxin. N. Engl. J. Med..

[11] Greene P.E., Fahn S. (1993). Use of botulinum toxin type F injections to treat torticollis in patients with immunity to botulinum toxin type A. Mov. Disord..

[12] Greene P.E., Fahn S. (1996). Response to botulinum toxin F in seronegative botulinum toxin A—Resistant patients. Mov. Disord..

[13] Mezaki T., Kaji R., Kohara N., Fujii H., Katayama M., Shimizu T., Kimura J., Brin M. (1995). Comparison of Therapeutic Efficacies of Type A and F Botulinum Toxins for Blepharospasm A double-blind, controlled study. Neurology.

[14] Houser M., Sheean G., Lees A. (1998). Further studies using higher doses of botulinum toxin type F for torticollis resistant to botulinum toxin type A. J. Neurol. Neurosurg. Psychiatry.

[15] Sheean G., Lees A. (1995). Botulinum toxin F in the treatment of torticollis clinically resistant to botulinum toxin A. J. Neurol. Neurosurg. Psychiatry.

[16] Chen R., Karp B.I., Hallett M. (1998). Botulinum toxin type F for treatment of dystonia Long-term experience. Neurology.

[17] Billante C.R., Zealear D.L., Billante M., Reyes J.H., Sant’Anna G., Rodriguez R., Stone R. (2002). Comparison of neuromuscular blockade and recovery with botulinum toxins A and F. Muscle Nerve.

[18] Eleopra R., Tugnoli V., Rossetto O., De Grandis D., Montecucco C. (1998). Different time courses of recovery after poisoning with botulinum neurotoxin serotypes A and E in humans. Neurosci. Lett..

[19] Adler M., Keller J.E., Sheridan R.E., Deshpande S.S. (2001). Persistence of botulinum neurotoxin A demonstrated by sequential administration of serotypes A and E in rat EDL muscle. Toxicon.

[20] Whitemarsh R.C.M., Tepp W.H., Johnson E.A., Pellett S. (2014). Persistence of botulinum neurotoxin a subtypes 1–5 in primary rat spinal cord cells. PLoS ONE.

[21] Antonucci F., Rossi C., Gianfranceschi L., Rossetto O., Caleo M. (2008). Long-distance retrograde effects of botulinum neurotoxin A. J. Neurosci..

[22] Scheps D., de la Paz M.L., Jurk M., Hofmann F., Frevert J. (2017). Design of modified botulinum neurotoxin A1 variants with a shorter persistence of paralysis and duration of action. Toxicon.

[23] Costantin L., Bozzi Y., Richichi C., Viegi A., Antonucci F., Funicello M., Gobbi M., Mennini T., Rossetto O., Montecucco C. (2005). Antiepileptic effects of botulinum neurotoxin E. J. Neurosci..

[24] Eleopra R., Tugnoli V., Rossetto O., Montecucco C., De Grandis D. (1997). Botulinum neurotoxin serotype C: A novel effective botulinum toxin therapy in human. Neurosci. Lett..

[25] Eleopra R., Tugnoli V., Quatrale R., Gastaldo E., Rossetto O., De Grandis D., Montecucco C. (2002). Botulinum neurotoxin serotypes A and C do not affect motor units survival in humans: An electrophysiological study by motor units counting. Clin. Neurophysiol..

[26] Eleopra R., Tugnoli V., De Grandis D., Montecucco C. (1998). Botulinum toxin serotype C treatment in subjects affected by focal dystonia and resistant to botulinum toxin serotype A. Neurology.

[27] Eleopra R., Tugnoli V., Quatrale R., Rossetto O., Montecucco C., Dressler D. (2006). Clinical use of non-A botulinum toxins: Botulinum toxin type C and botulinum toxin type F. Neurotox. Res..

[28] Pellett S., Tepp W.H., Whitemarsh R.C., Bradshaw M., Johnson E.A. (2015). In vivo onset and duration of action varies for botulinum neurotoxin A subtypes 1–5. Toxicon.

[29] Whitemarsh R.C., Tepp W.H., Bradshaw M., Lin G., Pier C.L., Scherf J.M., Johnson E.A., Pellett S. (2013). Characterization of botulinum neurotoxin A subtypes 1 through 5 by investigation of activities in mice, in neuronal cell cultures, and in vitro. Infect. Immun..

[30] Pier C.L., Chen C., Tepp W.H., Lin G., Janda K.D., Barbieri J.T., Pellett S., Johnson E.A. (2011). Botulinum neurotoxin subtype A2 enters neuronal cells faster than subtype A1. FEBS Lett..

[31] Akaike N., Shin M.C., Wakita M., Torii Y., Harakawa T., Ginnaga A., Kato K., Kaji R., Kozaki S. (2013). Transsynaptic inhibition of spinal transmission by A2 botulinum toxin. J. Physiol..

[32] Torii Y., Kiyota N., Sugimoto N., Mori Y., Goto Y., Harakawa T., Nakahira S., Kaji R., Kozaki S., Ginnaga A. (2011). Comparison of effects of botulinum toxin subtype A1 and A2 using twitch tension assay and rat grip strength test. Toxicon.

[33] Kroken A.R., Blum F.C., Zuverink M., Barbieri J.T. (2017). Entry of Botulinum neurotoxin subtypes A1 and A2 into neurons. Infect. Immun..

[34] Benoit R.M., Schärer M.A., Wieser M.M., Li X., Frey D., Kammerer R.A. (2017). Crystal structure of the BoNT/A2 receptor-binding domain in complex with the luminal domain of its neuronal receptor SV2C. Sci. Rep..

[35] Tepp W.H., Lin G., Johnson E.A. (2012). Purification and characterization of a novel subtype A3 botulinum neurotoxin. Appl. Environ. Microbiol..

[36] Torii Y., Goto Y., Nakahira S., Kozaki S., Ginnaga A. (2014). Comparison of the immunogenicity of botulinum toxin type A and the efficacy of A1 and A2 neurotoxins in animals with A1 toxin antibodies. Toxicon.

[37] Mukai Y., Shimatani Y., Sako W., Asanuma K., Nodera H., Sakamoto T., Izumi Y., Kohda T., Kozaki S., Kaji R. (2014). Comparison between botulinum neurotoxin type A2 and type A1 by electrophysiological study in healthy individuals. Toxicon.

[38] Kaji R. (2015). Clinical differences between A1 and A2 botulinum toxin subtypes. Toxicon.

[39] Itakura M., Kohda T., Kubo T., Semi Y., Azuma Y.-T., Nakajima H., Kozaki S., Takeuchi T. (2014). Botulinum neurotoxin A subtype 2 reduces pathological behaviors more effectively than subtype 1 in a rat Parkinson’s disease model. Biochem. Biophys. Res. Commun..

[40] Shin M.-C., Yukihira T., Ito Y., Akaike N. (2013). Antinociceptive effects of A1 and A2 type botulinum toxins on carrageenan-induced hyperalgesia in rat. Toxicon.

[41] Ma L., Nagai J., Sekino Y., Goto Y., Nakahira S., Ueda H. (2012). Single Application of A2 NTX, a Botulinum Toxin A2 Subunit, Prevents Chronic Pain Over Long Periods in Both Diabetic and Spinal Cord Injury–Induced Neuropathic Pain Models. J. Pharmacol. Sci..

[42] Debono D.J., Hoeksema L.J., Hobbs R.D. (2013). Caring for patients with chronic pain: Pearls and pitfalls. J. Am. Osteopath. Assoc..

[43] Van Hecke O., Torrance N., Smith B.H. (2013). Chronic pain epidemiology—Where do lifestyle factors fit in?. Br. J. Pain.

[44] Reid K.J., Harker J., Bala M.M., Truyers C., Kellen E., Bekkering G.E., Kleijnen J. (2011). Epidemiology of chronic non-cancer pain in Europe: Narrative review of prevalence, pain treatments and pain impact. Curr. Med. Res. Opin..

[45] Woolf C.J. (2010). Overcoming obstacles to developing new analgesics. Nat. Med..

[46] Kissin I. (2010). The development of new analgesics over the past 50 years: A lack of real breakthrough drugs. Anesth. Analg..

[47] Tarsy D., First E.R. (1999). Painful cervical dystonia: Clinical features and response to treatment with botulinum toxin. Mov. Disord..

[48] Freund B., Schwartz M. (2003). Temporal relationship of muscle weakness and pain reduction in subjects treated with botulinum toxin A. J. Pain.

[49] Bomba-Warczak E., Vevea J.D., Brittain J.M., Figueroa-Bernier A., Tepp W.H., Johnson E.A., Yeh F.L., Chapman E.R. (2016). Interneuronal Transfer and Distal Action of Tetanus Toxin and Botulinum Neurotoxins A and D in Central Neurons. Cell Rep..

[50] Cocco A., Albanese A. (2017). Recent developments in clinical trials of bont. Toxicon.

[51] Caleo M., Restani L. (2017). Direct central nervous system effects of botulinum neurotoxin. Toxicon.

[52] Marinelli S., Vacca V., Ricordy R., Uggenti C., Tata A.M., Luvisetto S., Pavone F. (2012). The analgesic effect on neuropathic pain of retrogradely transported botulinum neurotoxin A involves Schwann cells and astrocytes. PLoS ONE.

[53] Silva L.B.D., Poulsen J.N., Arendt-Nielsen L., Gazerani P. (2015). Botulinum neurotoxin type A modulates vesicular release of glutamate from satellite glial cells. J. Cell. Mol. Med..

[54] Zychowska M., Rojewska E., Makuch W., Luvisetto S., Pavone F., Marinelli S., Przewlocka B., Mika J. (2016). Participation of pro-and anti-nociceptive interleukins in botulinum toxin A-induced analgesia in a rat model of neuropathic pain. Eur. J. Pharmacol..

[55] Aurora S., Dodick D.W., Turkel C., DeGryse R., Silberstein S., Lipton R., Diener H., Brin M. (2010). OnabotulinumtoxinA for treatment of chronic migraine: Results from the double-blind, randomized, placebo-controlled phase of the PREEMPT 1 trial. Cephalalgia.

[56] Silberstein S.D., Dodick D.W., Aurora S.K., Diener H.-C., DeGryse R.E., Lipton R.B., Turkel C.C. (2015). Per cent of patients with chronic migraine who responded per onabotulinumtoxinA treatment cycle: PREEMPT. J. Neurol. Neurosurg. Psychiatry.

[57] Simpson D.M., Hallett M., Ashman E.J., Comella C.L., Green M.W., Gronseth G.S., Armstrong M.J., Gloss D., Potrebic S., Jankovic J. (2016). Practice guideline update summary: Botulinum neurotoxin for the treatment of blepharospasm, cervical dystonia, adult spasticity, and headache Report of the Guideline Development Subcommittee of the American Academy of Neurology. Neurology.

[58] Relja M., Telarović S. (2004). Botulinum toxin in tension-type headache. J. Neurol..

[59] Padberg M., De Bruijn S., De Haan R., Tavy D. (2004). Treatment of chronic tension-type headache with botulinum toxin: A double-blind, placebo-controlled clinical trial. Cephalalgia.

[60] Schulte-Mattler W.J., Krack P., Group B.S. (2004). Treatment of chronic tension-type headache with botulinum toxin A: A randomized, double-blind, placebo-controlled multicenter study. Pain.

[61] Silberstein S.D., Göbel H., Jensen R., Elkind A.H., Degryse R., Walcott J.M., Turkel C. (2006). Botulinum toxin type A in the prophylactic treatment of chronic tension-type headache: A multicentre, double-blind, randomized, placebo-controlled, parallel-group study. Cephalalgia.

[62] Khenioui H., Houvenagel E., Catanzariti J.F., Guyot M.A., Agnani O., Donze C. (2016). Usefulness of intra-articular botulinum toxin injections. A systematic review. Jt. Bone Spine.

[63] Heikkilä H., Hielm-Björkman A., Morelius M., Larsen S., Honkavaara J., Innes J., Laitinen-Vapaavuori O. (2014). Intra-articular botulinum toxin A for the treatment of osteoarthritic joint pain in dogs: A randomized, double-blinded, placebo-controlled clinical trial. Vet. J..

[64] Vilhegas S., Cassu R., Barbero R., Crociolli G., Rocha T., Gomes D. (2015). Botulinum toxin type A as an adjunct in postoperative pain management in dogs undergoing radical mastectomy. Vet. Rec..

[65] Mittal S., Machado D.G., Jabbari B. (2012). OnabotulinumtoxinA for treatment of focal cancer pain after surgery and/or radiation. Pain Med..

[66] Lacković Z., Filipović B., Matak I., Helyes Z. (2016). Activity of botulinum toxin type A in cranial dura: Implications for treatment of migraine and other headaches. Br. J. Pharmacol..

[67] Drinovac Vlah V., Filipović B., Bach-Rojecky L., Lacković Z. (2017). Role of central versus peripheral opioid system in antinociceptive and anti-inflammatory effect of botulinum toxin type A in trigeminal region. Eur. J. Pain.

[68] Nelson A.E. (2018). Osteoarthritis year in review 2017: Clinical. Osteoarthr. Cartil..

[69] Kidd B.L. (2006). Osteoarthritis and joint pain. Pain.

[70] Martell-Pelletier J., Barr A.J., Cicuttini F.M., Conaghan P.G., Cooper C., Goldring M.B., Goldring S.R., Jones G., Teichthal A.J., Pelletier J. (2016). Osteoarthritis. Nat. Rev. Dis. Prim..

[71] Ivanusic J.J. (2017). Molecular Mechanisms That Contribute to Bone Marrow Pain. Front. Neurol..

[72] Schaible H.-G., Ebersberger A., Von Banchet G.S. (2002). Mechanisms of Pain in Arthritis. Ann. N. Y. Acad. Sci..

[73] Hsieh L.-F., Wu C.-W., Chou C.-C., Yang S.-W., Wu S.-H., Lin Y.-J., Hsu W.-C. (2016). Effects of botulinum toxin landmark-guided intra-articular injection in subjects with knee osteoarthritis. PM&R.

[74] Arendt-Nielsen L., Jiang G., DeGryse R., Turkel C. (2017). Intra-articular onabotulinumtoxinA in osteoarthritis knee pain: Effect on human mechanistic pain biomarkers and clinical pain. Scand. J. Rheumatol..

[75] Krug H.E., Frizelle S., McGarraugh P., Mahowald M.L. (2009). Pain behavior measures to quantitate joint pain and response to neurotoxin treatment in murine models of arthritis. Pain Med..

[76] Anderson S., Krug H., Dorman C., McGarraugh P., Frizelle S., Mahowald M. (2010). Analgesic effects of intra-articular botulinum toxin Type B in a murine model of chronic degenerative knee arthritis pain. J. Pain Res..

[77] Wang L., Wang K., Chu X., Li T., Shen N., Fan C., Niu Z., Zhang X., Hu L. (2017). Intra-articular injection of Botulinum toxin A reduces neurogenic inflammation in CFA-induced arthritic rat model. Toxicon.

[78] Yoo K.Y., Lee H.S., Cho Y.K., Lim Y.S., Kim Y.S., Koo J.H., Yoon S.J., Lee J.H., Jang K.H., Song S.H. (2014). Anti-inflammatory Effects of Botulinum Toxin Type A in a Complete Freund’s Adjuvant-Induced Arthritic Knee Joint of Hind Leg on Rat Model. Neurotox. Res..

[79] Chapple C. (2014). Chapter 2: Pathophysiology of neurogenic detrusor overactivity and the symptom complex of “overactive bladder”. Neurourol. Urodyn..

[80] Merrill L., Gonzalez E.J., Girard B.M., Vizzard M.A. (2016). Receptors, channels, and signalling in the urothelial sensory system in the bladder. Nat. Rev. Urol..

[81] Traini C., Fausssone-Pellegrini M.S., Guasti D., Del Popolo G., Frizzi J., Serni S., Vannucchi M.G. (2018). Adaptive changes of telocytes in the urinary bladder of patients affected by neurogenic detrusor overactivity. J. Cell. Mol. Med..

[82] Schurch B., Stohrer M., Kramer G., Schmid D.M., Gaul G., Hauri D. (2000). Botulinum-A toxin for treating detrusor hyperreflexia in spinal cord injured patients: A new alternative to anticholinergic drugs? Preliminary results. J. Urol..

[83] Khera M., Somogyi G.T., Kiss S., Boone T.B., Smith C.P. (2004). Botulinum toxin A inhibits ATP release from bladder urothelium after chronic spinal cord injury. Neurochem. Int..

[84] Collins V.M., Daly D.M., Liaskos M., McKay N.G., Sellers D., Chapple C., Grundy D. (2013). OnabotulinumtoxinA significantly attenuates bladder afferent nerve firing and inhibits ATP release from the urothelium. BJU Int..

[85] Lucioni A., Bales G.T., Lotan T.L., McGehee D.S., Cook S.P., Rapp D.E. (2008). Botulinum toxin type A inhibits sensory neuropeptide release in rat bladder models of acute injury and chronic inflammation. BJU Int..

[86] Jhang J.F., Kuo H.C. (2016). Botulinum Toxin A and Lower Urinary Tract Dysfunction: Pathophysiology and Mechanisms of Action. Toxins (Basel).

[87] Giannantoni A., Conte A., Farfariello V., Proietti S., Vianello A., Nardicchi V., Santoni G., Amantini C. (2013). Onabotulinumtoxin-A intradetrusorial injections modulate bladder expression of NGF, TrkA, p75 and TRPV1 in patients with detrusor overactivity. Pharmacol. Res..

[88] Loiseau C., Iezhova T., Valkiunas G., Chasar A., Hutchinson A., Buermann W., Smith T.B., Sehgal R.N. (2010). Spatial variation of haemosporidian parasite infection in African rainforest bird species. J. Parasitol..

[89] Maignel-Ludop J., Huchet M., Krupp J. (2017). Botulinum Neurotoxins Serotypes A and B induce paralysis of mouse striated and smooth muscles with different potencies. Pharmacol. Res. Perspect..

[90] Coelho A., Dinis P., Pinto R., Gorgal T., Silva C., Silva A., Silva J., Cruz C.D., Cruz F., Avelino A. (2010). Distribution of the high-affinity binding site and intracellular target of botulinum toxin type A in the human bladder. Eur. Urol..

[91] Coelho A., Oliveira R., Cruz F., Cruz C.D. (2016). Impairment of sensory afferents by intrathecal administration of botulinum toxin A improves neurogenic detrusor overactivity in chronic spinal cord injured rats. Exp. Neurol..

[92] Eming S.A., Martin P., Tomic-Canic M. (2014). Wound repair and regeneration: Mechanisms, signaling, and translation. Sci. Transl. Med..

[93] Lebeda F.J., Dembek Z.F., Adler M. (2012). Kinetic and reaction pathway analysis in the application of botulinum toxin A for wound healing. J. Toxicol..

[94] Ziade M., Domergue S., Batifol D., Jreige R., Sebbane M., Goudot P., Yachouh J. (2013). Use of botulinum toxin type A to improve treatment of facial wounds: A prospective randomised study. JPRAS.

[95] Prodromidou A., Frountzas M., Vlachos D.-E.G., Vlachos G.D., Bakoyiannis I., Perrea D., Pergialiotis V. (2015). Botulinum toxin for the prevention and healing of wound scars: A systematic review of the literature. Plast. Surg..

[96] Lee B.-J., Jeong J.-H., Wang S.-G., Lee J.-C., Goh E.-K., Kim H.-W. (2009). Effect of botulinum toxin type a on a rat surgical wound model. Clin. Exp. Otorhinolaryngol..

[97] Kiritsi D., Nyström A. (2017). The role of TGFβ in wound healing pathologies. Mech. Ageing Dev..

[98] Xiao Z., Zhang F., Lin W., Zhang M., Liu Y. (2010). Effect of botulinum toxin type A on transforming growth factor beta1 in fibroblasts derived from hypertrophic scar: A preliminary report. Aesth. Plast. Surg..

[99] Xiao Z., Zhang M., Liu Y., Ren L. (2011). Botulinum toxin type a inhibits connective tissue growth factor expression in fibroblasts derived from hypertrophic scar. Aesth. Plast. Surg..

[100] Pirazzini M., Rossetto O., Eleopra R., Montecucco C. (2017). Botulinum Neurotoxins: Biology, Pharmacology, and Toxicology. Pharmacol. Rev..

[101] Popoff M.R., Poulain B. (2010). Bacterial toxins and the nervous system: Neurotoxins and multipotential toxins interacting with neuronal cells. Toxins (Basel).

[102] Poulain B. (2008). How do the Botulinum Neurotoxins block neurotransmitter release: From botulism to the molecular mechanism of action. Botulinum J..

[103] Rossetto O. (2018). Botulinum Toxins: Molecular Structures and Synaptic Physiology. Botulinum Toxin Treat. Clin. Med..

[104] Rossetto O., Pirazzini M., Montecucco C. (2014). Botulinum neurotoxins: Genetic, structural and mechanistic insights. Nat. Rev. Microbiol..

[105] Rummel A. (2015). The long journey of botulinum neurotoxins into the synapse. Toxicon.

[106] Matak I., Lackovic Z., Relja M. (2016). Botulinum toxin type A in motor nervous system: Unexplained observations and new challenges. J. Neural Transm. (Vienna).

[107] Mazzocchio R., Caleo M. (2015). More than at the neuromuscular synapse: Actions of botulinum neurotoxin A in the central nervous system. Neuroscientist.

[108] Kanovsky P., Streitova H., Dufek J., Znojil V., Daniel P., Rektor I. (1998). Change in lateralization of the P22/N30 cortical component of median nerve somatosensory evoked potentials in patients with cervical dystonia after successful treatment with botulinum toxin A. Mov. Disord..

[109] Delnooz C.C., Pasman J.W., van de Warrenburg B.P. (2015). Dynamic cortical gray matter volume changes after botulinum toxin in cervical dystonia. Neurobiol. Dis..

[110] Behari M., Raju G.B. (1996). Electrophysiological studies in patients with blepharospasm before and after botulinum toxin A therapy. J. Neurol. Sci..

[111] Bielamowicz S., Ludlow C.L. (2000). Effects of botulinum toxin on pathophysiology in spasmodic dysphonia. Ann. Otol. Rhinol. Laryngol..

[112] Marchand-Pauvert V., Aymard C., Giboin L.S., Dominici F., Rossi A., Mazzocchio R. (2013). Beyond muscular effects: Depression of spinal recurrent inhibition after botulinum neurotoxin A. J. Physiol..

[113] Aymard C., Giboin L.S., Lackmy-Vallee A., Marchand-Pauvert V. (2013). Spinal plasticity in stroke patients after botulinum neurotoxin A injection in ankle plantar flexors. Physiol. Rep..

[114] Clowry G.J., Walker L., Davies P. (2006). The effects of botulinum neurotoxin A induced muscle paresis during a critical period upon muscle and spinal cord development in the rat. Exp. Neurol..

[115] Gonzalez-Forero D., Pastor A.M., Geiman E.J., Benitez-Temino B., Alvarez F.J. (2005). Regulation of gephyrin cluster size and inhibitory synaptic currents on Renshaw cells by motor axon excitatory inputs. J. Neurosci..

[116] Cai B.B., Francis J., Brin M.F., Broide R.S. (2017). Botulinum neurotoxin type A-cleaved SNAP25 is confined to primary motor neurons and localized on the plasma membrane following intramuscular toxin injection. Neuroscience.

[117] Koizumi H., Goto S., Okita S., Morigaki R., Akaike N., Torii Y., Harakawa T., Ginnaga A., Kaji R. (2014). Spinal Central Effects of Peripherally Applied Botulinum Neurotoxin A in Comparison between Its Subtypes A1 and A2. Front. Neurol..

[118] Matak I., Riederer P., Lackovic Z. (2012). Botulinum toxin’s axonal transport from periphery to the spinal cord. Neurochem. Int..

[119] Filipovic B., Matak I., Bach-Rojecky L., Lackovic Z. (2012). Central action of peripherally applied botulinum toxin type A on pain and dural protein extravasation in rat model of trigeminal neuropathy. PLoS ONE.

[120] Restani L., Giribaldi F., Manich M., Bercsenyi K., Menendez G., Rossetto O., Caleo M., Schiavo G. (2012). Botulinum neurotoxins A and E undergo retrograde axonal transport in primary motor neurons. PLoS Pathog..

[121] Wu C., Xie N., Lian Y., Xu H., Chen C., Zheng Y., Chen Y., Zhang H. (2016). Central antinociceptive activity of peripherally applied botulinum toxin type A in lab rat model of trigeminal neuralgia. Springerplus.

[122] Restani L., Novelli E., Bottari D., Leone P., Barone I., Galli-Resta L., Strettoi E., Caleo M. (2012). Botulinum neurotoxin A impairs neurotransmission following retrograde transynaptic transport. Traffic.

[123] Wang T., Martin S., Papadopulos A., Harper C.B., Mavlyutov T.A., Niranjan D., Glass N.R., Cooper-White J.J., Sibarita J.B., Choquet D. (2015). Control of autophagosome axonal retrograde flux by presynaptic activity unveiled using botulinum neurotoxin type A. J. Neurosci..

[124] Harper C.B., Papadopulos A., Martin S., Matthews D.R., Morgan G.P., Nguyen T.H., Wang T., Nair D., Choquet D., Meunier F.A. (2016). Botulinum neurotoxin type-A enters a non-recycling pool of synaptic vesicles. Sci. Rep..

[125] Lawrence G.W., Ovsepian S.V., Wang J., Aoki K.R., Dolly J.O. (2012). Extravesicular intraneuronal migration of internalized botulinum neurotoxins without detectable inhibition of distal neurotransmission. Biochem. J..

[126] Ramachandran R., Lam C., Yaksh T.L. (2015). Botulinum toxin in migraine: Role of transport in trigemino-somatic and trigemino-vascular afferents. Neurobiol. Dis..

[127] Bentivoglio A.R., Fasano A., Ialongo T., Soleti F., Lo Fermo S., Albanese A. (2009). Fifteen-year experience in treating blepharospasm with Botox or Dysport: Same toxin, two drugs. Neurotox. Res..

[128] Dong Y., Wu T., Hu X., Wang T. (2017). Efficacy and safety of botulinum toxin type A for upper limb spasticity after stroke or traumatic brain injury: A systematic review with meta-analysis and trial sequential analysis. Eur. J. Phys. Rehabil. Med..

[129] Gu H.Y., Song J.K., Zhang W.J., Xie J., Yao Q.S., Zeng W.J., Zhang C., Niu Y.M. (2017). A systematic review and meta-analysis of effectiveness and safety of therapy for overactive bladder using botulinum toxin A at different dosages. Oncotarget.

[130] Naumann M., Jankovic J. (2004). Safety of botulinum toxin type A: A systematic review and meta-analysis. Curr. Med. Res. Opin..

[131] Shaari C.M., George E., Wu B.L., Biller H.F., Sanders I. (1991). Quantifying the spread of botulinum toxin through muscle fascia. Laryngoscope.

[132] Yaraskavitch M., Leonard T., Herzog W. (2008). Botox produces functional weakness in non-injected muscles adjacent to the target muscle. J. Biomech..

[133] Arezzo J.C. (2009). NeuroBloc/Myobloc: Unique features and findings. Toxicon.

[134] Borodic G.E., Joseph M., Fay L., Cozzolino D., Ferrante R.J. (1990). Botulinum A toxin for the treatment of spasmodic torticollis: Dysphagia and regional toxin spread. Head Neck.

[135] Carli L., Montecucco C., Rossetto O. (2009). Assay of diffusion of different botulinum neurotoxin type a formulations injected in the mouse leg. Muscle Nerve.

[136] Hsu T.S., Dover J.S., Arndt K.A. (2004). Effect of volume and concentration on the diffusion of botulinum exotoxin A. Arch. Dermatol..

[137] Wohlfarth K., Schwandt I., Wegner F., Jurgens T., Gelbrich G., Wagner A., Bogdahn U., Schulte-Mattler W. (2008). Biological activity of two botulinum toxin type A complexes (Dysport and Botox) in volunteers: A double-blind, randomized, dose-ranging study. J. Neurol..

[138] Brodsky M.A., Swope D.M., Grimes D. (2012). Diffusion of botulinum toxins. Tremor Other Hyperkinet. Mov..

[139] Bakheit A.M., Ward C.D., McLellan D.L. (1997). Generalised botulism-like syndrome after intramuscular injections of botulinum toxin type A: A report of two cases. J. Neurol. Neurosurg. Psychiatry.

[140] Bhatia K.P., Munchau A., Thompson P.D., Houser M., Chauhan V.S., Hutchinson M., Shapira A.H., Marsden C.D. (1999). Generalised muscular weakness after botulinum toxin injections for dystonia: A report of three cases. J. Neurol. Neurosurg. Psychiatry.

[141] Ramirez-Castaneda J., Jankovic J., Comella C., Dashtipour K., Fernandez H.H., Mari Z. (2013). Diffusion, spread, and migration of botulinum toxin. Mov. Disord..

[142] Aoki R.K. (2002). Botulinum neurotoxin serotypes A and B preparations have different safety margins in preclinical models of muscle weakening efficacy and systemic safety. Toxicon.

[143] Torii Y., Goto Y., Takahashi M., Ishida S., Harakawa T., Sakamoto T., Kaji R., Kozaki S., Ginnaga A. (2010). Quantitative determination of biological activity of botulinum toxins utilizing compound muscle action potentials (CMAP), and comparison of neuromuscular transmission blockage and muscle flaccidity among toxins. Toxicon.

[144] Johnson E.A., Tepp W.H., Lin G. (2013). Purification, Characterization, and Use of Clostridium Botulinum Neurotoxin BoNT/A3. Patent.

[145] Torii Y., Goto Y., Nakahira S., Kozaki S., Kaji R., Ginnaga A. (2015). Comparison of systemic toxicity between botulinum toxin subtypes A1 and A2 in mice and rats. Basic Clin. Pharmacol. Toxicol..

[146] Schantz E.J., Johnson E.A. (1992). Properties and Use of Botulinum Toxin and Other Microbial Neurotoxins in Medicine. Microbiol. Rev..

[147] Frokjaer S., Otzen D.E. (2005). Protein drug stability: A formulation challenge. Nature.

[148] Ascher B., Kestemont P., Boineau D., Bodokh I., Stein A., Heckmann M., Dendorfer M., Pavicic T., Volteau M., Tse A. (2018). Liquid Formulation of AbobotulinumtoxinA Exhibits a Favorable Efficacy and Safety Profile in Moderate to Severe Glabellar Lines: A Randomized, Double-Blind, Placebo- and Active Comparator-Controlled Trial. Aesthet. Surg. J..

[149] Jankovic J. (2004). Botulinum toxin in clinical practice. J. Neurol. Neurosurg. Psychiatry.

[150] Bagramyan K., Barash J.R., Arnon S.S., Kalkum M. (2008). Attomolar Detection of Botulinum Toxin Type A in Complex Biological Matrices. PLoS ONE.

[151] Mason J.T., Xu L., Sheng Z.-M., He J., O’Leary T.J. (2006). Liposome polymerase chain reaction assay for the sub-attomolar detection of cholera toxin and botulinum toxin type A. Nat. Protocol..

[152] Prausnitz M.R., Langer R. (2008). Transdermal drug delivery. Nat. Biotechnol..

[153] Waugh J.M., Lee J., Dake M.D., Browne D. (2011). Nonclinical and clinical experiences with CPP-based self-assembling peptide systems in topical drug development. Cell Penetr. Pept. Methods Protocol..

[154] Jones T., Jeremy Scott C., Tranowski D., Joshi T. Safety and Tolerability of Topical Botulinum Toxin Type A in Healthy Adults. Proceedings of the 69th Annual Meeting of the Society for Investigative Dermatology.

[155] Brandt F., O’connell C., Cazzaniga A., Waugh J.M. (2010). Efficacy and safety evaluation of a novel botulinum toxin topical gel for the treatment of moderate to severe lateral canthal lines. Dermatol. Surg..

[156] Glogau R., Brandt F., Kane M., Monheit G.D., Waugh J.M. (2012). Results of a randomized, double-blind, placebo-controlled study to evaluate the efficacy and safety of a botulinum toxin type A topical gel for the treatment of moderate-to-severe lateral canthal lines. J. Drugs Dermatol..

[157] Glogau R.G. (2007). Topically applied botulinum toxin type A for the treatment of primary axillary hyperhidrosis: Results of a randomized, blinded, vehicle-controlled study. Dermatol. Surg..

[158] Chajchir I., Modi P., Chajchir A. (2008). Novel topical BoNTA (CosmeTox, toxin type A) cream used to treat hyperfunctional wrinkles of the face, mouth, and neck. Aesthet. Plast. Surg..

[159] Carmichael N.M., Dostrovsky J.O., Charlton M.P. (2010). Peptide-mediated transdermal delivery of botulinum neurotoxin type A reduces neurogenic inflammation in the skin. Pain.

[160] Saffarian P., Peerayeh S.N., Amani J., Ebrahimi F., Sedighian H., Halabian R., Fooladi A.A.I. (2016). TAT-BoNT/A (1–448), a novel fusion protein as a therapeutic agent: Analysis of transcutaneous delivery and enzyme activity. Appl. Microbiol. Biotechnol..

[161] Saffarian P., Peerayeh S.N., Amani J., Ebrahimi F., Sedighianrad H., Halabian R., Imani Fooladi A.A. (2016). Expression and purification of recombinant TAT-BoNT/A (1–448) under denaturing and native conditions. Bioengineered.

[162] Chow A., Wilder-Smith E. (2009). Effect of transdermal botulinum toxin on sweat secretion in subjects with idiopathic palmar hyperhidrosis. Br. J. Dermatol..

[163] Kavanagh G.M., Shams K. (2006). Botulinum toxin type A by iontophoresis for primary palmar hyperhidrosis. J. Am. Acad. Dermatol..

[164] Pacini S., Gulisano M., Punzi T., Ruggiero M. (2007). Transdermal delivery of Clostridium botulinum toxin type A by pulsed current iontophoresis. J. Am. Acad. Dermatol..

[165] Iannitti T., Palmieri B., Aspiro A., Di Cerbo A. (2014). A preliminary study of painless and effective transdermal botulinum toxin A delivery by jet nebulization for treatment of primary hyperhidrosis. Drug Des. Dev. Ther..

[166] Bariya S.H., Gohel M.C., Mehta T.A., Sharma O.P. (2012). Microneedles: An emerging transdermal drug delivery system. J. Pharm. Pharmacol..

[167] Torrisi B.M., Zarnitsyn V., Prausnitz M., Anstey A., Gateley C., Birchall J.C., Coulman S. (2013). Pocketed microneedles for rapid delivery of a liquid-state botulinum toxin A formulation into human skin. J. Control. Release.

[168] Tyagi P., Kashyap M., Yoshimura N., Chancellor M., Chermansky C.J. (2017). Past, Present and Future of Chemodenervation with Botulinum Toxin in the Treatment of Overactive Bladder. J. Urol..

[169] Khera M., Somogyi G.T., Salas N.A., Kiss S., Boone T.B., Smith C.P. (2005). In vivo effects of botulinum toxin A on visceral sensory function in chronic spinal cord-injured rats. Urology.

[170] Shimizu S., Wheeler M., Saito M., Weiss R., Hittelman A. (2012). 907 Effect of intravesical botulinum toxin a delivery (using dmso) in rat overactive bladder model. J. Urol..

[171] Petrou S.P., Parker A.S., Crook J.E., Rogers A., Metz-Kudashick D., Thiel D.D. (2009). Botulinum a toxin/dimethyl sulfoxide bladder instillations for women with refractory idiopathic detrusor overactivity: A phase 1/2 study. Mayo Clin. Proc..

[172] Vemulakonda V.M., Somogyi G.T., Kiss S., Salas N.A., Boone T.B., Smith C.P. (2005). Inhibitory effect of intravesically applied botulinum toxin A in chronic bladder inflammation. J. Urol..

[173] Sweeney D., O’Leary M., Erickson J., Marx S., Chancellor M. Safety and efficacy with bladder botulinum toxin in elderly patients. Proceedings of the International Continence Society Annual Meeting.

[174] Chuang Y.-C., Yoshimura N., Huang C.-C., Wu M., Chiang P.-H., Chancellor M.B. (2009). Intravesical botulinum toxin A administration inhibits COX-2 and EP4 expression and suppresses bladder hyperactivity in cyclophosphamide-induced cystitis in rats. Eur. Urol..

[175] El Shatoury M., Di Young L., Turley E., Yazdani A., Dave S. (2017). Early experimental results of using a novel delivery carrier, hyaluronan-phosphatidylethanolamine (HA-PE), which may allow simple bladder instillation of botulinum toxin A as effectively as direct detrusor muscle injection. J. Pediatr. Urol..

[176] Krhut J., Navratilova M., Sykora R., Jurakova M., Gärtner M., Mika D., Pavliska L., Zvara P. (2016). Intravesical instillation of onabotulinum toxin A embedded in inert hydrogel in the treatment of idiopathic overactive bladder: A double-blind randomized pilot study. Scand. J. Urol..

[177] Chuang Y.-C., Tyagi P., Huang C.-C., Yoshimura N., Wu M., Kaufman J., Chancellor M.B. (2009). Urodynamic and immunohistochemical evaluation of intravesical botulinum toxin A delivery using liposomes. J. Urol..

[178] Chuang Y.-C., Kaufmann J.H., Chancellor D.D., Chancellor M.B., Kuo H.-C. (2014). Bladder instillation of liposome encapsulated onabotulinumtoxina improves overactive bladder symptoms: A prospective, multicenter, double-blind, randomized trial. J. Urol..

[179] Chuang Y.-C., Huang T.-L., Tyagi P., Huang C.-C. (2016). Urodynamic and immunohistochemical evaluation of intravesical botulinum toxin A delivery using low energy shock waves. J. Urol..

[180] Kajbafzadeh A.-M., Montaser-Kouhsari L., Ahmadi H., Sotoudeh M. (2011). Intravesical electromotive botulinum toxin type A administration: Part I—Experimental study. Urology.

[181] Schiotz H.A., Mai H.T., Zabielska R. (2017). Intravesical Electromotive Botulinum Toxin in Women with Overactive Bladder—A Pilot Study. ARC J. Gynecol. Obs..

[182] Kajbafzadeh A.-M., Ahmadi H., Montaser-Kouhsari L., Sharifi-Rad L., Nejat F., Bazargan-Hejazi S. (2011). Intravesical electromotive botulinum toxin type A administration—Part II: Clinical application. Urology.

[183] Ladi-Seyedian S.-S., Sharifi-Rad L., Kajbafzadeh A.-M. (2017). Intravesical Electromotive Botulinum Toxin Type “A” Administration for Management of Urinary Incontinence Secondary to Neuropathic Detrusor Overactivity in Children: Long-Term Follow-up. Urology.

[184] Kajbafzadeh A.-M., Sharifi-Rad L., Ladi-Seyedian S.-S. (2016). Intravesical electromotive botulinum toxin type A administration for management of concomitant neuropathic bowel and bladder dysfunction in children. Int. J. Colorectal Dis..

[185] Zhu Z., Stone H.F., Thach T.Q., Garcia L., Ruegg C.L. (2012). A novel botulinum neurotoxin topical gel: Treatment of allergic rhinitis in rats and comparative safety profile. Am. J. Rhinol. Allergy.

[186] Rohrbach S., Junghans K., Köhler S., Laskawi R. (2009). Minimally invasive application of botulinum toxin A in patients with idiopathic rhinitis. Head Face Med..

